# New naphthalene-containing enamides: synthesis, structural insights and biological screening as potential anticancer agents against Huh-7 cancer cell line

**DOI:** 10.1039/d5ra07730c

**Published:** 2025-12-16

**Authors:** Rasha Mohammed Saleem, Arwa Sultan Alqahtani, Rabah N. Alsulami, Islam Zaki

**Affiliations:** a Department of Laboratory Medicine, Faculty of Applied Medical Sciences, Al-Baha University Al-Baha 65431 Saudi Arabia; b Department of Chemistry, College of Science, Imam Mohammad Ibn Saud Islamic University (IMSIU) P. O. Box 90950 Riyadh 11623 Saudi Arabia; c Department of Biology, College of Applied Sciences, Umm Al-Qura University Makkah Saudi Arabia; d Pharmaceutical Organic Chemistry Department, Faculty of Pharmacy, Port Said University Port Said Egypt Eslam.Zaki@pharm.psu.edu.eg

## Abstract

Naphthalene and enamide derivatives are prevalent intracellular tubulin assembly inhibitors, and their optimization is critical for the creation of targeted anticancer agents. A number of new compounds containing naphthalen-1-yloxy and enamide functions connected through *N*′-acetoxyhydrazide linker were designed and synthesized and their biological activity were tested as possible anticancer agents. The cytotoxic activity of the constructed naphthalene–enamide analogs was studied on Huh-7 hepatocellular carcinoma cell line. The analogs 5f and 5g with 4-methylbenzene and 4-methoxybenzene in the 3,4,5-trimethoxyenamide moiety were proved to have outstanding cytotoxic activity with superior cytotoxic action (IC_50_ = 2.62 and 3.37 µM, respectively) toward the growth of Huh-7 cells compared to conventional anticancer agent Dox (IC_50_ = 7.20 µM). In addition, the most efficient members had strong inhibitory efficacy against tubulin beta polymerization. Additionally, the most potent analog had cellular cycle arrest at G2/M phase while lowering the cellular population at G1 and S phases relative to controls. Fluorochrome Annexin-V and PI FACS staining assessment disclosed that Huh-7 hatched with compound 5f elevated the percentage of total apoptosis compared to untreated controls. Furthermore, compound 5f had a strong pro-apoptotic impact through triggering the intrinsic mechanism of apoptosis. This mechanistic route was verified *via* FACS experiment that indicated a remarkable drop in the extent of MMP compared with the controls.

## Abstract 2

A number of new compounds containing naphthalen-1-yloxy and enamide functions connected through *N*′-acetoxyhydrazide linker were designed, synthesized and their biological activity were tested as possible anticancer agents.

## Graphical abstract

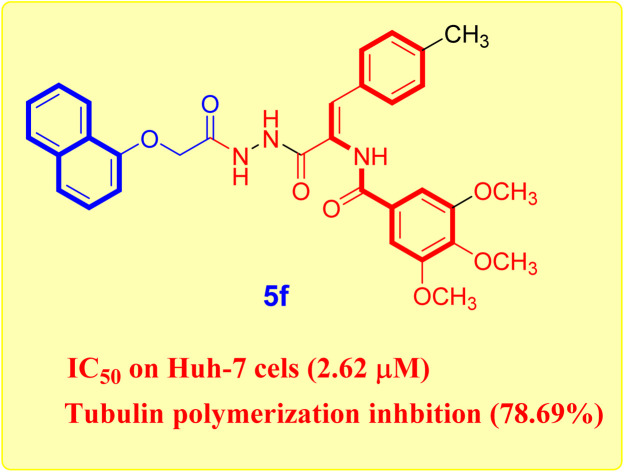

## 1. Introduction

Cancer is considered as one of the most serious health threats worldwide.^1^ This may be due to the annual rise in cases as well as the high death rate, which ranks cardiovascular disease as the second leading cause of death.^2^ The greater danger of this disease may result from its severity and the malignant cells' propensity to withstand therapy with traditional chemotherapeutic agents.^3^ Therefore, the search for a novel anticancer drug with high potency and low toxicity is a hotspot of ongoing research on novel drugs to combat this resistance.^4^

**Fig. 1:**
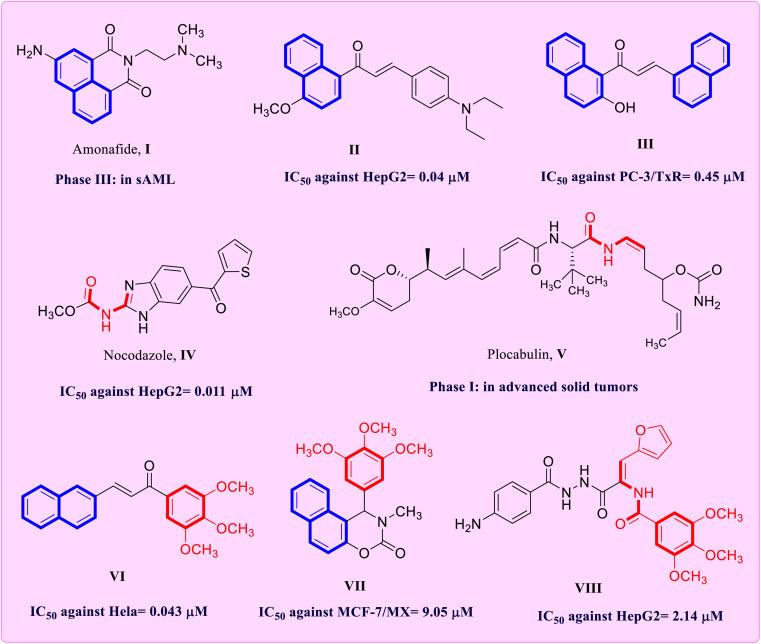
Structures of representative naphthalene and enamide derivatives I–VIII.

Naphthalene-containing scaffolds are an appealing template due to their remarkable therapeutic attributes, which have been reported in numerous antiproliferative compounds.^5–7^ For instance amonafide (naphthylimide) I a drug in phase III clinical trials for treatment of secondary acute myeloid leukemia (sAML).^8^ In addition, the naphthalene–enone derivative II showed strong cytotoxic activity and inhibited tubulin polymerization of HepG2 hepatocellular carcinoma cells at the IC_50_ concentration (0.04 µM).^9^ Additionally, compound III (1,3-dinaphthyl chalcone) showed noteworthy antiproliferative effects against taxane-resistant prostate cancer (PC-3/TxR) cell line and exhibited apoptotic cellular death^10^ (Fig. 1).

Moreover, the enamide structure is a key pharmacophore in pharmaceutical chemistry, serving as building blocks for new drug candidates.^11,12^ The widespread use as scaffold places enamide in the class of privileged structures.^13^ Enamide is incorporated in a range of biological activities.^14^ Specifically, a number of substituted enamides have been shown to have anticancer activity.^15^ Enamides containing compounds have been proposed to disrupt the cell cycle disruption and cause apoptosis, inhibit tubulin growth and affect specific kinases required for cancer cell growth and survival.^16–18^ Nocodazole IV is defined as microtubule-inhibiting agent that interferes with the assembly of microtubules.^19^ In addition, plocabulin V a drug currently in the first phase of clinical trials for the management of advanced solid tumors with multi-target actions including inhibition of microtubule development, their dynamics and antiangiogenic abilities^20^ (Fig. 1).

The well-known tubulin protein is essential for many cellular functions, particularly spindle formation and chromosomal segregation during cell division.^21^ Tubulin polymerization inhibitors are becoming a well-established approach for the generation of highly efficient antimitotic drugs for cancer treatment.^22–24^ The colchicine binding site inhibitors (CSIs), as the most often investigated target for antimitotic drugs, have become one of the most successful targets in the creation of chemotherapeutic drugs.^25–27^ A literature search for functional groups that might be used as pharmacophores for the anticancer therapy found that many CSIs share the 3,4,5-trimethoxyphenyl (TMP) moiety.^28^ Some naphthalene and enamide derivatives with a TMP moiety, such as compounds VI–VIII have demonstrated strong tubulin polymerization inhibition behavior.^29–31^ These compounds suppress cell division by targeting the colchicine-binding site, preventing of tubulin polymerization, stopping G/M phase cell cycle progression and causing apoptosis.^32^ These facts confirm the significance of CSIs as an efficient therapeutic target for cancer management.

Molecular hybridization is a technique that combines two or more active pharmacophores to generate single hybrid analogue with synergistic biological attributes, which is used to develop new anticancer drug candidates for complex disorders.^33^ Hybrid molecules are designed to boost the potency and biological spectrum of the parent molecules, overcome drug cross resistance and lowered potential toxicity relative to the parent drugs.^34^ In view of above mentioned facts and an attempt to achieve extremely effective anticancer agents, it was contemplated to describe the synthesis of a series of certain novel naphthalene–enamide derivatives 4a,b and 5a–i that incorporate naphthalene and substituted enamide functions into a single compacted structure for synergistic anticancer activity with the prime mission of developing effective anticancer agents. The amide fragment of enamide function is decorated with dimethoxyphenyl (DMP) or trimethoxyphenyl (TMP) moiety as (H-bond) acceptor–donor (A–D) pair forming group to reinforce the interaction with target protein. In addition, the alkenyl fragment of enamide function is equipped with substituted phenyl group or heterocyclic moiety such as furan. Further, the substitution pattern on the phenyl moiety was chosen so as to assure different electronic and lipophilic attributes which should influence the activity of the target analogs. The synthesized analogs were evaluated for cytotoxic attributes against hepatocellular cell line (Huh-7). Such cell line was chosen since they demonstrated increased microtubule formation.^35^ The active compounds were endured to flow cytometry analysis of DNA content for cellular cycle, apoptosis and migration assay in Huh-7. In addition their impacts on mitochondrial membrane potential and safety profile on normal cell line were also reported. The results of the *in vitro* assessments demonstrated that the synthesized analogs may serve as valuable anticancer tubulin beta polymerization inhibitors.

## 2. Results and discussion

### 2.1. Chemistry

**Scheme 1:**
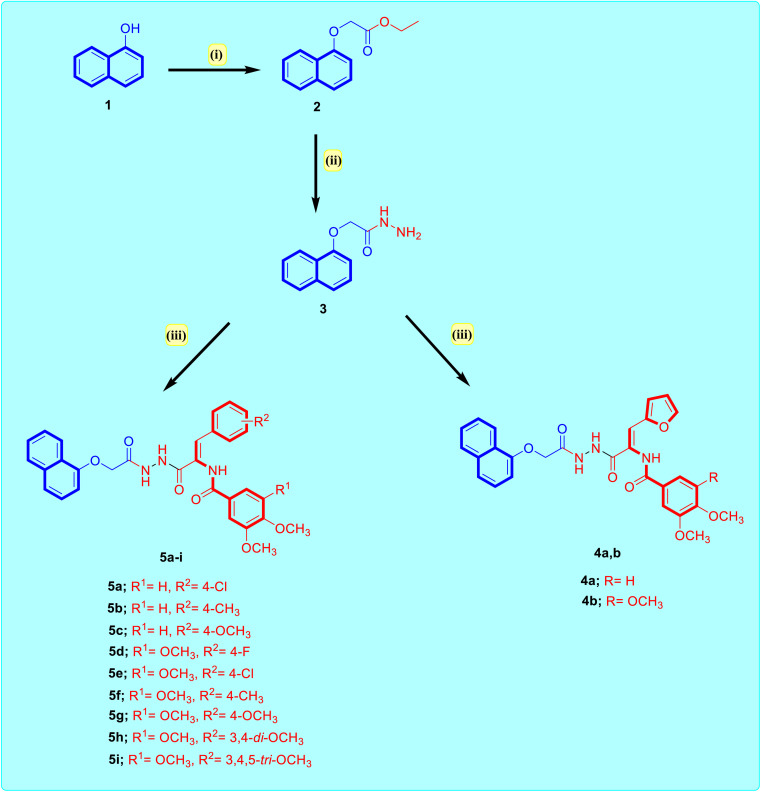
Synthesis of the target naphthalene–enamide analogs 4a,b and 5a–i. Reagents: (i) ClCH_2_COOEt, K_2_CO_3_, acetone, reflux 24 h, 88%; (ii) NH_2_NH_2_.H_2_O, EtOH, reflux 8 h, 91%; (iii) respective ethyl 3-aryl-2-arylamidoprop-2-enoate, EtOH, AcOH, reflux 10–12 h, 68–79%.

The target naphthalene–enamide derivatives were synthesized from starting material, α-naphthol in a sequence of chemical reactions, depicted in Scheme 1. First, α-naphthol reacted with ethyl chloroacetate in pure acetone including anhydrous potassium carbonate to give ethyl naphthalen-1-yloxyacetate 2.^36^ The ester compound 2 on condensation with pure hydrazine in pure ethanol awarded naphthalen-1-yloxyacetohydrazide 3.^37^ Finally, naphthalen-1-yloxyacetohydrazide 3 reacted with appropriate enamide ester in pure ethanol containing few drops of acetic acid under reflux. The reaction mixture was poured into ice-cold water which afforded the title naphthalene–enamide analogs 4a,b and 5a–i in good to excellent yields (68–79%). The newly synthesized naphthalene–enamide analogs were purified by crystallization technique with DMF/pure ethanol (3 : 1) solvent mixture. The structures of target naphthalene–enamide analogs were authenticated on the basis of their respective ^1^H-NMR and ^13^C-NMR spectra as well as elemental microanalysis. The ^1^H-NMR spectra of the resulting products were consistent with their respective structures. In the ^1^H-NMR spectrum of 5f as representative example, three singlet peaks were prominent and appeared at *δ*_H_ 10.38, 9.99 and 8.37 ppm related to (NH) protons, also, the existence of two protons of C8–H and C5–H at *δ*_H_ 8.37 and 7.89, respectively, of the 1-naphthyl moiety in the form of doublet peaks. In addition, ^1^H-NMR spectrum of analog 5d conspicuously manifested peak at *δ*_H_ 7.25 ppm with one proton integration compatible with enamide proton, besides, ^1^H-NMR spectrum indicated the chemical shift of *δ*_H_ 4.86 with two protons corresponding to (O–CH_2_–C=O) as well as two singlet peaks with three and six protons integration at *δ*_H_ 3.87 and 3.75 ppm, respectively ascribed to trimethoxyphenyl functions of enamide moiety.

^13^C-NMR spectrum of naphthalene–enamide 5d revealed the presence of three peaks at *δ*_C_ 172.49, 167.12 and 165.82 ppm corresponding to the carbon atoms of amide moieties. Also, ^13^C-NMR spectrum indicated the chemical shifts of the methoxy carbons at *δ*_C_ 60.58 and 56.55 ppm as well as different carbon peaks all matching with the predictable structure. The enamide and aromatic carbons were observed in the range at *δ* 164.78–106.13 ppm and were in agreement with the respective molecular formula. The percentage of CHN analyses was well within the permissible limits.

### 2.2. Biological profiling

#### 2.2.1. Cytotoxic activity evaluation against Huh-7 liver cancer cells

To study the antiproliferative activity of the newly constructed naphthalene–tethered enamide analogs 4a,b and 5a–i, the cytotoxic activity was assessed using liver (Huh-7) cancer cell line. The prepared naphthalene–enamide derivatives showed considerable cytotoxic activity on the examined cell line with IC_50_ range of 2.62–43.96 µM. The *p*-tolyl derivative 5f showed the series' most potent activity with IC_50_ value of 2.62 µM, and more potent than that of the reference standard Doxorubicin (Dox) which showed IC_50_ value of 7.20 µM. Additionally, the *p*-methoxyphenyl 5g was this series' second most potent with IC_50_ of 3.37 µM which is also more potent than Dox. Regarding the activity of 3,4-dimethoxyphenylenamide derivative 4a and 5a–c towards Huh-7 cell line, substitution with furan-2-yl (4a), 4-chlorophenyl (5a), 4-methylphenyl (5b) or 4-methoxyphenyl (5c) decreased the cytotoxic activity with IC_50_ ranges of 11.17–43.96 µM compared to the utilized reference standard Dox (IC_50_ = 7.20 µM). With respect to the antiproliferative activity of the 3,4,5-trimethoxyphenylenamide derivatives 4b and 5d–i against Huh-7 cell line, substitution with furan-2-yl (4b: IC_50_ = 6.53 µM), 4-methylphenyl (5g: IC_50_ = 2.62 µM) or 4-methoxyphenyl (5g: IC_50_ = 3.37 µM) enhanced the cytotoxic action relative to the utilized reference Dox (IC_50_ = 7.20 µM). Whereas substitution with halo-attached phenyl derivatives such as 4-fluorophenyl (5d) or 4-chlorophenyl (5e) reduced the level of activity with IC_50_ values of 19.08 and 21.83 µM, respectively. Additionally, the substitution of 3,4,5-trimethoxyenamide derivatives with polysubstituted phenyl moiety such as 3,4-dimethoxyphenyl (5h) or 3,4,5-trimethoxyphenyl (5i) maintained the level of activity with IC_50_ values of 6.89 and 16.93 µM, respectively (Table 1).

**Table 1:** IC_50_ ± SD (µM) of constructed naphthalene–enamide analogs 4a,b and 5a–i

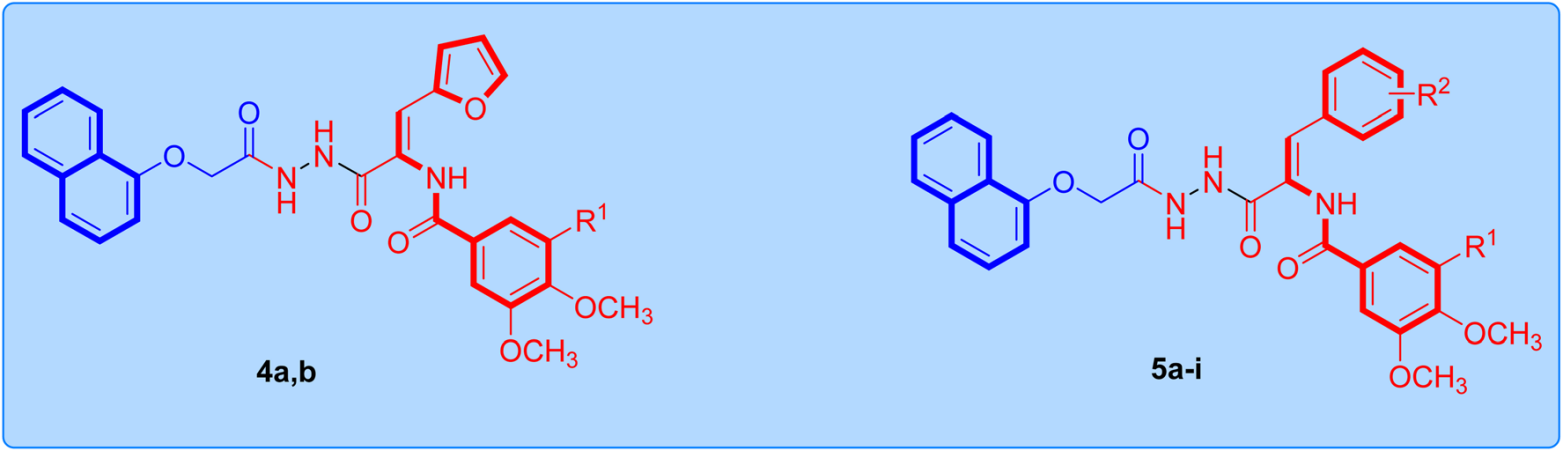			
Comp No	*R* ^1^	*R* ^2^	IC_50_ (µM)
			Huh-7
4a	–H	—	43.96 ± 1.83
4b	–OCH_3_	—	6.53 ± 0.22
5a	–H	4-Cl	28.72 ± 1.35
5b	–H	4-CH_3_	26.43 ± 1.44
5c	–H	4-OCH_3_	11.17 ± 0.37
5d	–OCH_3_	4 F	19.08 ± 0.61
5e	–OCH_3_	4-Cl	21.83 ± 0.69
5f	–OCH_3_	4-CH_3_	2.62 ± 0.12
5g	–OCH_3_	4-OCH_3_	3.37 ± 0.17
5h	–OCH_3_	3,4-di-OCH_3_	6.89 ± 0.26
5i	–OCH_3_	3,4,5-tri-OCH_3_	16.93 ± 0.57
Dox	—		7.20 ± 0.33

#### 2.2.2. Tubulin beta polymerization inhibition technique

**Fig. 2:**
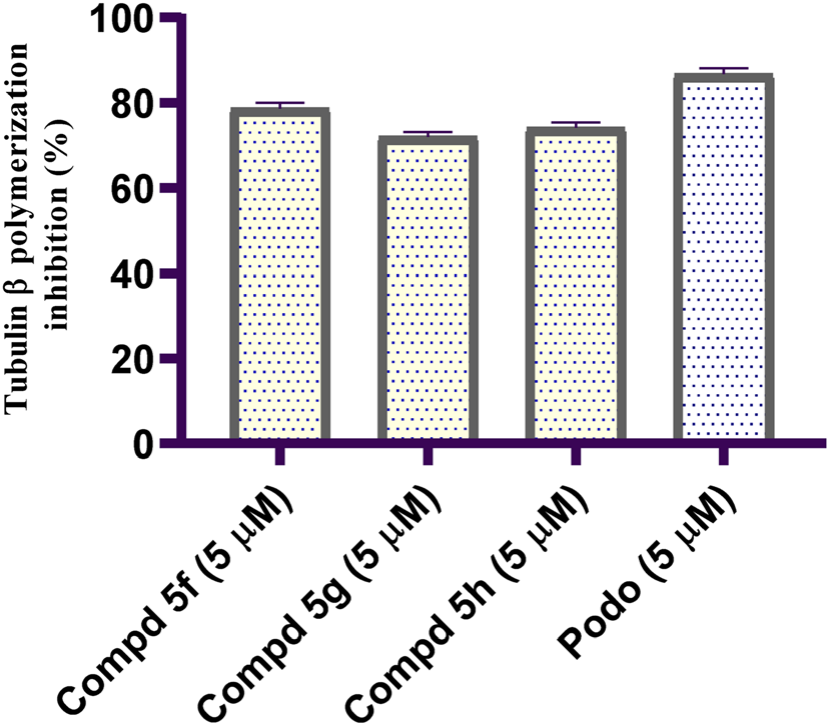
Graphical illustration of tubulin beta (TUBβ) polymerization inhibition percentage for naphthalene–tethered enamide analogs 5f, 5g and 5h compared with Podo.

Antimitotic drugs are among the most often utilized chemotherapeutic agents for metastatic liver cancer.^38^ These agents affect the dynamics of microtubules in the mitotic spindles by mainly binding to β-tubulin, a key protein in the mitotic spindle.^39^ Thus, blocking mitotic spindle assembly and interfering with sister chromatids' regular migration towards the spindle poles make for an appealing cancer treatment target.^40^ Accordingly, utilizing podophyllotoxin (Podo) as reference antimitotic drug, the tubulin beta (TUBβ) polymerization inhibitory contribution of the most potent naphthalene–enamide analogs was assessed in order to evaluate the significance of TUBβ polymerization on Huh-7 cells and to figure out the potential mode of the most cytotoxic analogs against investigated Huh-7 cells. This assay was applied for naphthalene–enamide analogs 5f, 5g and 5h which offered the most promise in contrast to Huh-7 cell line (IC_50_ = 2.62, 3.37 and 6.89 µM, respectively). Fig. 2 provides a summary of the tubulin beta polymerization inhibiting influences of the investigated naphthalene–enamides; 5f, 5g and 5h, expressed as percentage values. All three investigated naphthalene–enamide analogs demonstrated substantial TUBβ polymerization inhibition activity in line with the Huh-7 cell line's *in vitro* cytotoxic activity. Compound 5f showed significant TUBβ polymerization inhibition impact with percentage inhibition value of 78.69%. It was the most potent TUBβ polymerization inhibitor relative to the standard Podo drug (tubulin beta polymerization inhibition percentage = 86.67%).

#### 2.2.3. *In vitro* normal cell cytotoxicity

**Fig. 3:**
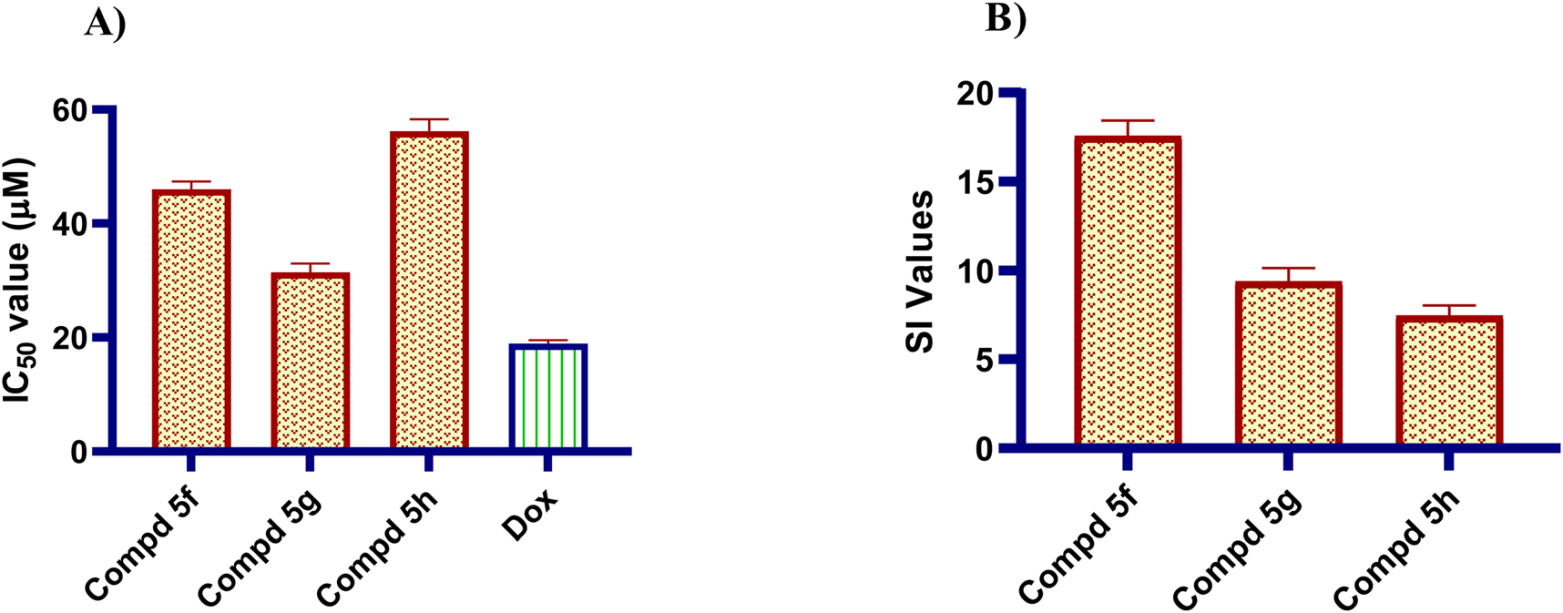
(A) IC_50_ ± SD (µM) of the most potent naphthalene–enamide derivatives 5f, 5g and 5h relative to Dox for IC_50_ determination against THLE-2 normal liver cell line. (B) Selectivity indices of the constructed analogs.

To assess the safety profile of the newly synthesized naphthalene–enamide analogs and their ability to be selective towards cancerous cells, the cytotoxic action of the most potent naphthalene–enamide analogs was examined on non-tumorigenic human liver cell line (THLE-2). All of the investigated analogs had a high IC_50_ on THLE-2 normal liver cell line comparing to their IC_50_ on cancer liver cell line Huh-7, with a selective index greater than 7.46-folds. The examined naphthalene–enamide analogs 5f, 5g and 5h showed IC_50_ on normal liver cells of 46.01, 31.60 and 51.43 µM, respectively (Fig. 3). These results found that naphthalene–enamide analogs were not only effective in inhibiting TUBβ polymerization and cytotoxicity, but also showed selectivity for cancerous cells, making them a safe and tolerable option for normal liver cells.

#### 2.2.4. Cell cycle analysis

**Fig. 4:**
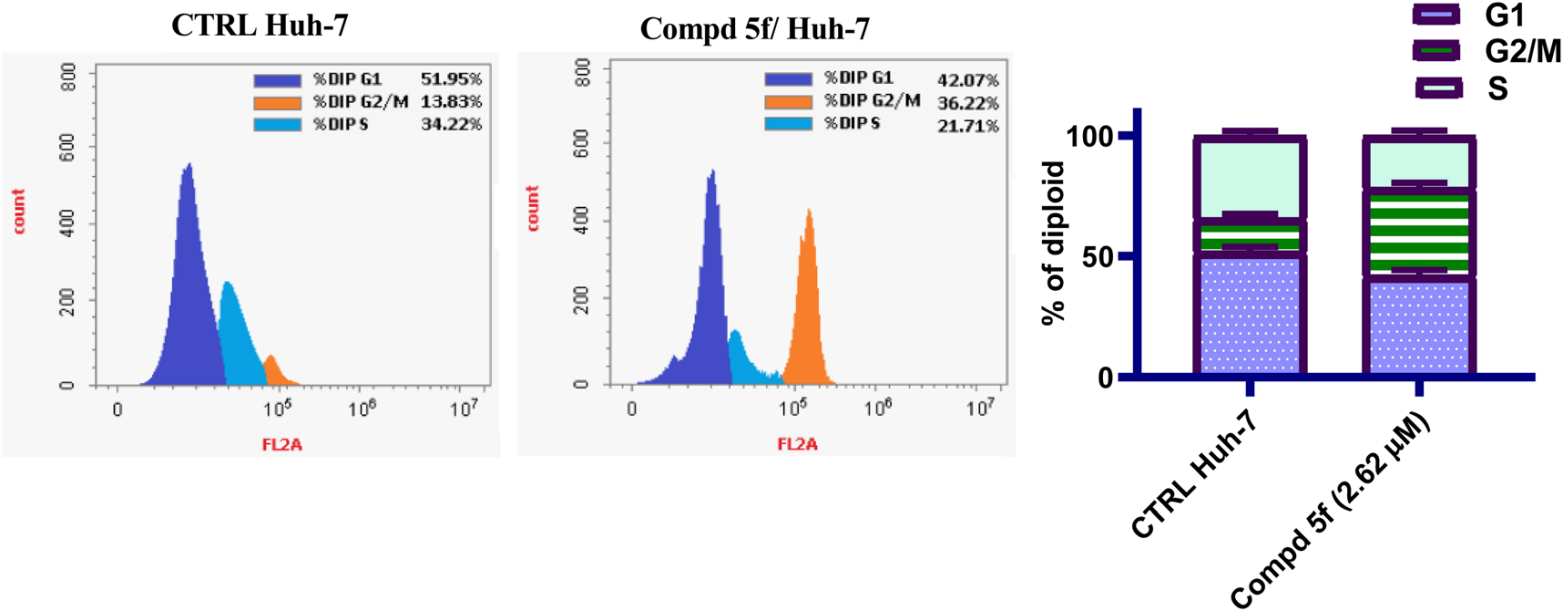
Cell cycle kinetics of Huh-7 liver cancer cells after treatment with naphthalene–enamide analog 5f.

In general, the majority of cells are dormant and don't divide unless prompted to enter the active stage of the cell cycle.^41^ This control is compromised or altered in a variety of illness, including cancer.^42^ In this case, it is critical to determine the status of the cell cycle phase and create treatments that specifically target aberrant cells.^43^ In order to determine the involvement of naphthalene–enamide analog 5f in growth suppression of cancerous cells, the impact of 5f on cell cycle stimulation of Huh-7 cancer cells was examined using DNA flow cytometric analysis. The tumor cells were managed with analog 5f at the IC_50_ concentration (µM), which were then stained with PI and quantified by flow cytometry. The results are shown in Fig. 4. A notable elevation in the cells at G2/M phase, 36.22% for analog 5f-treated cells was identified in comparison controls (G2/M phase = 13.83%). This has been accompanied by a diminution in the percentage cells at both G1 and S phases; 42.07 and 21.71%, respectively was identified with respect to control untreated groups (51.95 and 34.22%, respectively). From the results, compound 5f increased the accumulation of Huh-7 cells at G2/M phase with significant drop in the G1 phase and S phase relative to control. Thus it could be concluded that naphthalene–enamide 5f exerted antiproliferative activity against Huh-7 cells and tubulin beta polymerization inhibitory action by promoting cellular cycle arrest at G2/M phase.

#### 2.2.5. Apoptosis detection assay

**Fig. 5:**
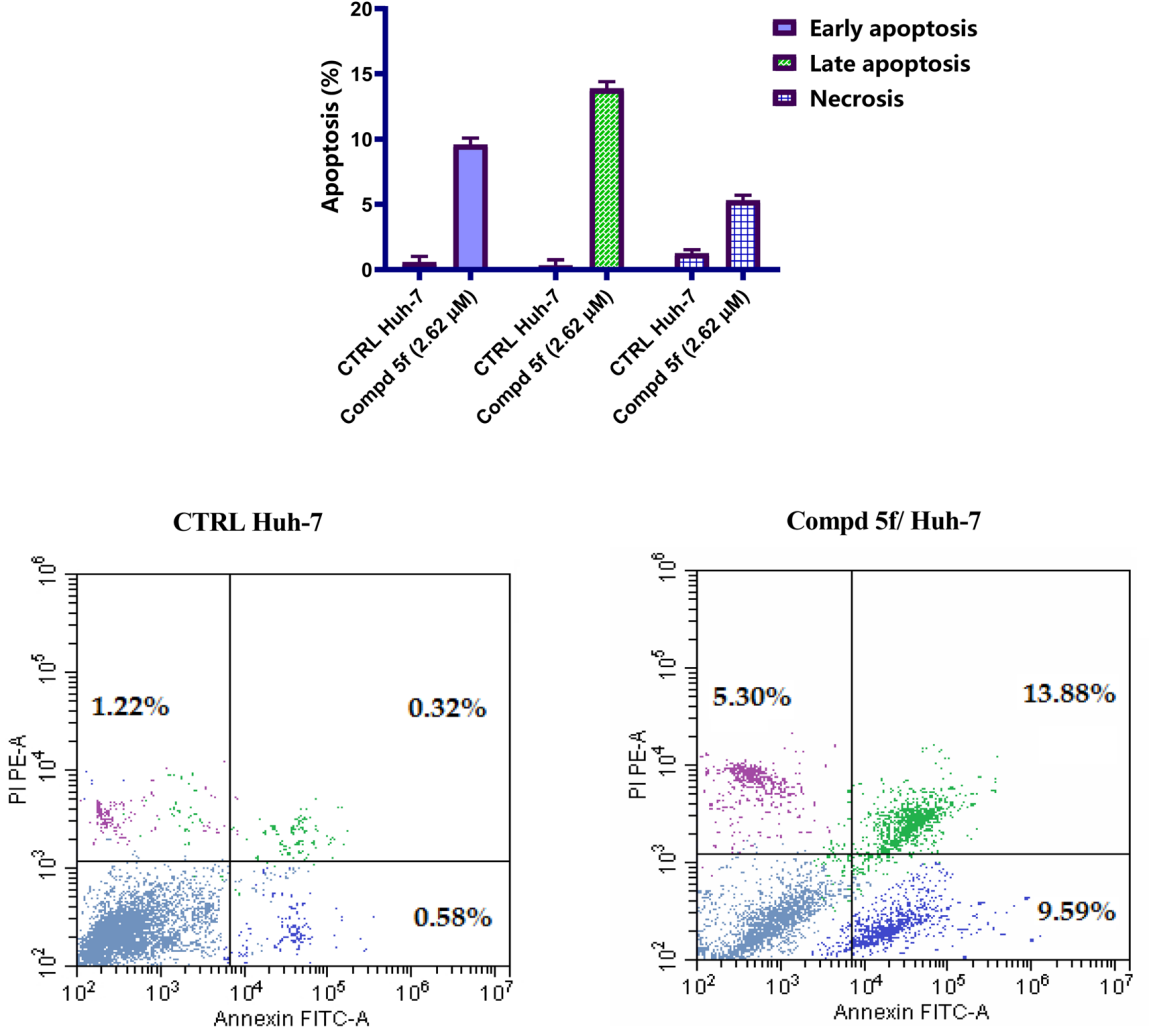
FITC Annexin-V and PI double staining after Huh-7 cells were hatched with naphthalene–enamide analog 5f at the IC_50_ (2.62 µM) concentration relative to control groups.

Significant evidences suggest that programmed death of cells, or apoptosis, is one of the fundamental pathways that safeguard the organisms from the emergence of cancer disease.^44^ Dysregulation of apoptosis can upset the delicate equilibrium between cellular death and proliferation, leading to illnesses such as cancer.^45^ For evaluating the process of apoptotic, induced apoptosis in Huh-7 cells by naphthalene–enamide compound 5f was assessed using propidium iodide (PI) and Annexin V-FITC staining assay.^46^ Huh-7 cells were hatched with IC_50_ concentration of naphthalene–enamide analog 5f for 48 h, then, the cells were marked with the two dyes. The associated red (PI) and green (FITC) fluorescence was identified with the flow cytometric analysis. In comparison to DMSO as negative control (Fig. 5), it was observed that naphthalene–enamide analog 5f induced an increase in the late/secondary cellular apoptosis from 0.32 (DMSO controls) to 13.88% for analog 5f-hatched Huh-7 cells. Also, a rise in the early/primary apoptosis was observed for naphthalene–enamide analog 5f-treated Huh-7 cells from 0.58 (DMSO controls) to 9.59%. The data corroborated the apoptosis contribution of naphthalene–enamide analog 5f.

#### 2.2.6. Measurement of mitochondrial membrane potential (ΔΨm)

**Fig. 6:**
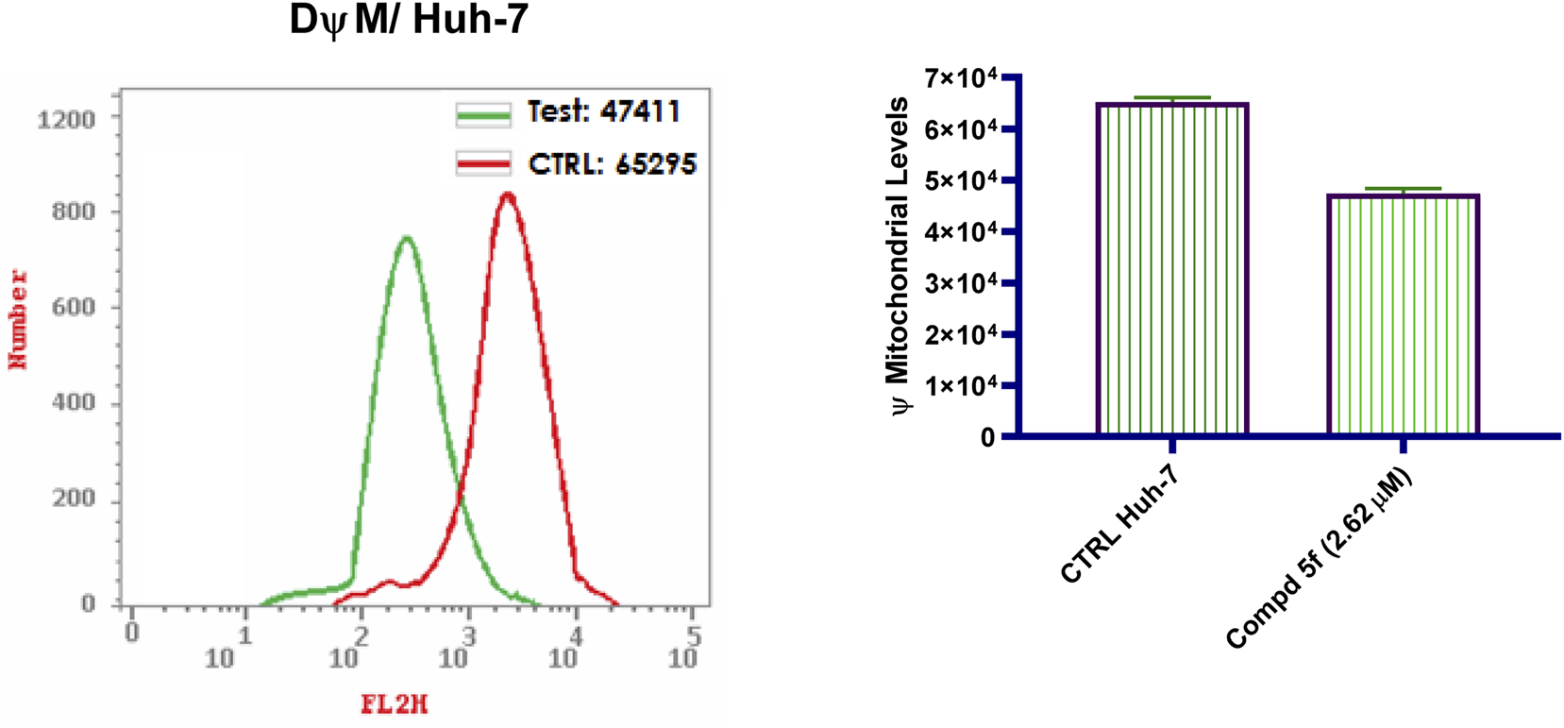
Flow cytometric measurements of the MMP following Huh-7 cells were hatched with naphthalene–enamide analog 5f at the IC_50_ (2.62 µM) concentration relative to control untreated Huh-7 cells.

Mitochondrial depolarization is one of the most common features of apoptosis-triggered cell death.^47^ Mitochondrial membrane potential (MMP) regulates the integrity of mitochondria and cellular bioenergetic functions.^48^ Recent research has shown that chemotherapeutic agents can provoke mitochondrial membrane depolarization and, as a result, promote apoptosis in a variety of cancer cells.^49^ By creating a pore or a voltage-dependent anion channel in the exterior mitochondrial membrane, mitochondria may facilitate the expulsion of cytochrome c.^50^ After it is in the cytoplasm, cytochrome c triggers apoptosis by activating Apaf-1, which in turn triggers procaspase-9, which triggers caspase 3.^51^ In this regard, the capacity of naphthalene–enamide analogs to elicit alterations in MMP of Huh-7 cells was explored following 48 h of treatment with compound 5f. In comparison to DMSO as negative controls (Fig. 6), it was observed that examined analog 5f lowered the level of MMP with percentage inhibition value of 72.61% for examined naphthalene-enamide 5f-treated Huh-7 cells. The results substantially indicated that the mitochondrial pathway is engaged in naphthalene–enamide 5f-induced Huh-7 cellular apoptosis.

#### 2.2.7. Effect of naphthalene–enamide 5f on Huh-7 cells migration

**Fig. 7:**
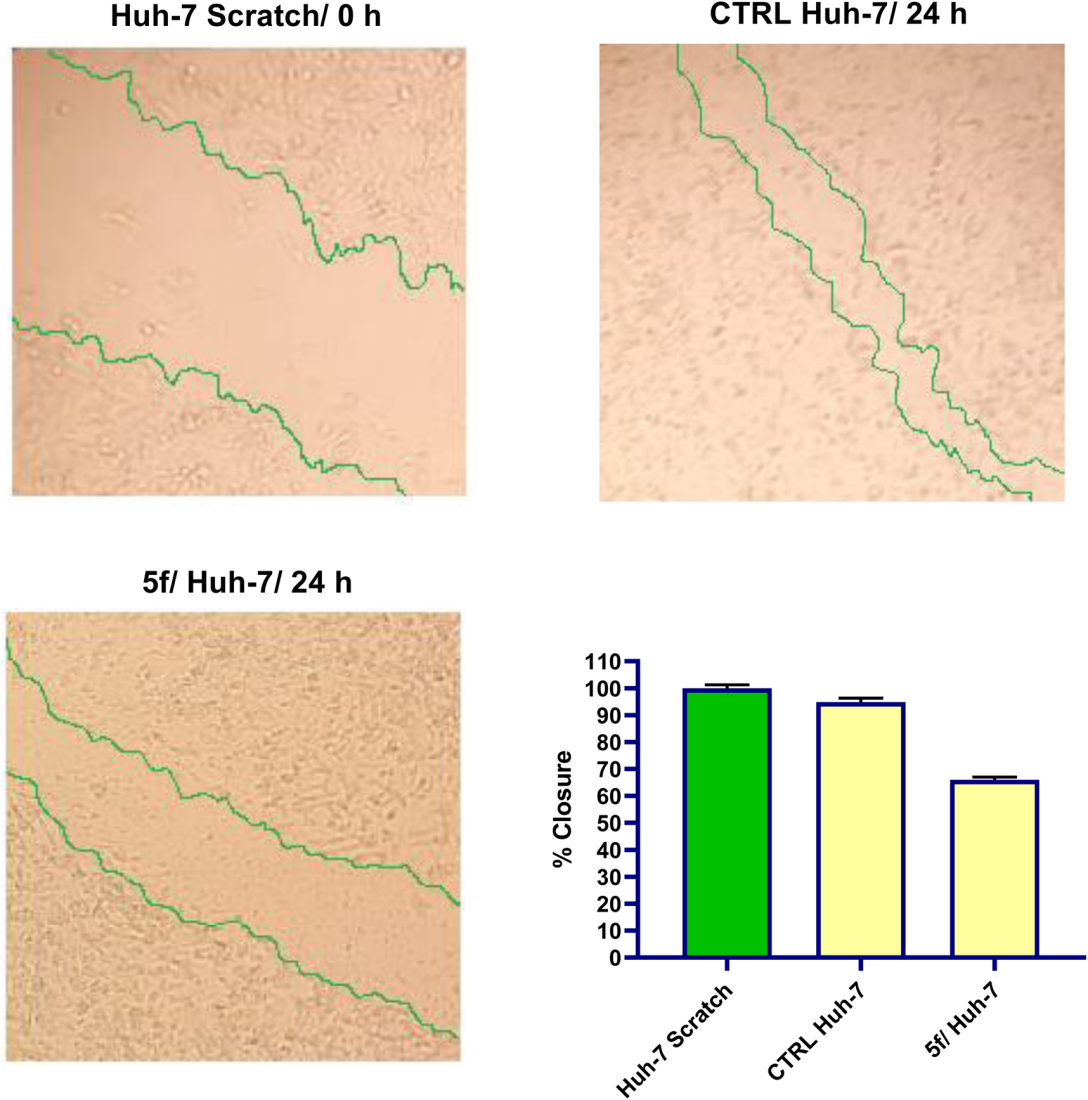
Influence of analog 5f on Huh-7 cells migration. Scratch wound healing experiment was utilized to assess the inhibition of cellular migration in Huh-7 cells treated for 24 h with analog 5f and the vehicle cells served as negative controls.

The *in vitro* scratch wound experiment was used to assess the hopeful of analog 5f to impede the ability of Huh-7 cells to migrate and heal.^52^ The main idea of this investigation is to produce a scratch in a monolayer of cancer cell line, and measure the diameter at the beginning and at regular intervals to evaluate the cancer cell' ability to migrate and heal.^53^ Next, the treated cell line's results are then contrasted with those of control untreated cell line.^54^Fig. 7 shows findings of scratch area at time points 0 and 24 h. After being exposed to Huh-7 treatment with synthetic naphthalene–enamide analog 5f for 24 h, the cell monolayer scratch showed partial closure in contrast to negative controls. Images taken at the ending of the 24 h incubation time confirmed that the untreated Huh-7 cells' monolayer scratch showed a 94.85% closure percentage. On the other hand, the percentage closure of the scratch in Huh-7 cell line treated with compound 5f at the IC_50_ concentration (µM) showed a modest drop from the control cell's scratch, which had a percentage closure of 65.93% as shown in Fig. 7. These findings demonstrated that analog 5f can substantially suppress Huh-7 cells migration and healing.

## 3. Conclusions

Certain new naphthalene derivatives 4a,b and 5a–i bearing enamide moiety have been synthesized and screened for their *in vitro* cytotoxic activity. The results revealed that elaborating naphthalene-tethered 3,4,5-trimethoxyphenylenamide moiety in the constructed molecules conferred the most active members, showing effectiveness toward Huh-7 hepatocellular carcinoma cell line. The analogs 5f and 5g possessed remarkable potent antiproliferative activity with IC_50_ values of 2.62 and 3.37 µM, respectively, superior to the reference drug Dox (IC_50_ = 7.20 µM). The possible mechanism behind the cytotoxic action of the promising compound 5f was thoroughly studied. Compound 5f can also be regarded as a lead for further development of anti-tubulin inhibitors, because it displayed significant tubulin beta polymerization () inhibitory activity. Furthermore, compound 5f raised the proportion of cells in the apoptotic stage and drove cell cycle arrest at G2/M phase. Moreover, the considerable rise in the percentage of both primary and secondary apoptotic cells indicated pro-apoptotic activity. Also, analog 5f caused a 72.61% inhibition of MMP compared with the untreated control cells, providing evidence that cancer cellular apoptosis was *via* mitochondrial-dependent route. Behind that, analog 5f inhibited migration in Huh-7 cancerous cells. Finally, analog 5f had minimal cytotoxic impact on normal hepatic cells with safer mode of action than the currently utilized anticancer agent Dox. So, analog 5f would become a valuable lead for an additional investigation. Also, substituted naphthalene moiety will be utilized as a starting material for further modification. In conclusion, successful applications of these results into healthcare settings could smooth the way for the development of alternative treatment approaches, ultimately benefiting patients of liver cancer and other diseases characterized by tubulin-microtubule polymerization abnormalities.

## 4. Experimental

### 4.1. Chemistry

#### 4.1.1. General method for the synthesis of (*Z*)-*N*-(1-ary1-3-(2-(2-(naphthalen-1-yloxy)acetyl)hydraziney1)-3-oxoprop-1-en-2-yl)arylamides 4a,b and 5a–i

Respective ethyl 3-aryl-2-arylamidoprop-2-enoate derivative (0.69 mmol) was added to a solution of naphthalene-1-yloxyacetohydrazide (0.69 mmol, 0.15 g) in pure ethanol (20 mL) while stirring till clear solution was obtained. A few drops of acetic acid were added as catalyst. Reflux was continued for 10–12 h (until complete consumption of ethyl 3-aryl-2-arylamidoprop-2-enoate molecule as monitored by TLC). After that, the reaction mixture was allowed to cool down to ambient temperature before being concentrated under reduced pressure. The residue thus obtained was treated with petroleum ether and crystallized from 10 mL of DMF/pure ethanol (3 : 1) applying mixed solvent recrystallization method to attain pure naphthalene–enamide derivatives 4a,b and 5a–i.

##### 4.1.1.1. (*Z*)-*N*-(1-(Furan-2-y1)-3-(2-(2-(naphthalen-1-yloxy)acetyl)hydraziney1)-3-oxoprop-1-en-2-y1)-3,4-dimethoxybenzamide (4a)

Light yellow powder, yield: (0.27 g, 75%), m.p 218–220 °C. IR (ATR, *ν*, cm^−1^): 3457, 3396, 3280 (NH), 3060, 3027 (CH aromatic), 2967, 2908 (CH aliphatic), 1712, 1680, 1630 (C=O), 1598, 1528 (C=C), 1296, 1125 (C–O). ^1^H-NMR (400 MHz, DMSO-*d*_6_) *δ*: 10.33 (s, 1H), 10.21 (s, 1H), 9.75 (s, 1H), 8.41–8.24 (m, 1H), 7.89 (dd, *J* = 7.1, 2.3 Hz, 1H), 7.79 (d, *J* = 1.8 Hz, 1H), 7.69 (dd, *J* = 8.5, 2.1 Hz, 1H), 7.64 (d, *J* = 2.1 Hz, 1H), 7.58–7.48 (m, 3H), 7.42 (t, *J* = 8.1 Hz, 1H), 7.20 (s, 1H), 7.09 (d, *J* = 8.7 Hz, 1H), 6.99 (d, *J* = 7.6 Hz, 1H), 6.74 (d, *J* = 3.5 Hz, 1H), 6.62 (ddd, *J* = 14.3, 3.5, 1.8 Hz, 1H), 4.83 (s, 2H), 3.85 (s, 6H). ^13^C-NMR (101 MHz, DMSO) *δ*: 167.05, 165.75, 164.35, 153.83, 152.12, 150.06, 148.63, 145.14, 134.49, 127.82, 127.01, 126.66, 126.55, 126.47, 125.75, 125.30, 122.52, 121.90, 121.15, 118.62, 114.67, 112.82, 111.80, 111.35, 106.15, 66.86, 56.14, 56.09. Elemental analysis for C_28_H_25_N_3_O_7_ (515.52): C‚ 65.24; H‚ 4.89; N‚ 8.15. Found: C‚ 65.32; H‚ 5.02; N‚ 8.00.

##### 4.1.1.2. (2)-*N*-(1-(Furan-2-y1)-3-(2-(2-(naphthalen-1-yloxy)acetyl)hydraziney1)-3-oxoprop-1-en-2-y1)-3,4,5-trimetboxybenzamide (4b)

Light yellow powder, yield: (0.29 g, 77%), m.p 214–216 °C. IR (ATR, *ν*, cm^−1^): 3473, 3306, 3235 (NH), 3081, 3056 (CH aromatic), 2959, 2934 (CH aliphatic), 1694, 1656, 1634 (C=O), 1599, 1586 (C=C), 1273, 1158 (C–O). ^1^H-NMR (400 MHz, DMSO-*d*_6_) *δ*: 10.33 (s, 1H), 10.21 (s, 1H), 9.83 (s, 1H), 8.40–8.23 (m, 1H), 7.93–7.87 (m, 1H), 7.81 (d, *J* = 1.7 Hz, 1H), 7.54 (ddd, *J* = 8.2, 5.6, 2.4 Hz, 3H), 7.45–7.42 (m, 1H), 7.40 (d, *J* = 3.0 Hz, 2H), 7.22 (s, 1H), 6.99 (d, *J* = 7.7 Hz, 1H), 6.77 (d, *J* = 3.5 Hz, 1H), 6.61 (dd, *J* = 3.5, 1.8 Hz, 1H), 4.83 (s, 2H), 3.87 (s, 6H), 3.75 (s, 3H). ^13^C-NMR (101 MHz, DMSO) *δ*: 167.06, 165.68, 164.24, 153.82, 153.00, 149.98, 145.31, 140.80, 134.49, 129.42, 127.82, 127.01, 126.47, 126.36, 125.75, 125.29, 122.52, 121.15, 118.81, 114.97, 112.86, 106.15, 106.08, 66.87, 60.58, 56.54. Elemental analysis for C_29_H_27_N_3_O_8_ (545.55): C‚ 63.85; H‚ 4.99; N‚ 7.70. Found: C‚ 64.03; H‚ 5.08; N‚ 7.56.

##### 4.1.1.3. (*Z*)-*N*-(1-(4-Chloropheny1)-3-(2-(2-(naphthalen-1-yloxy)acetyl)hydrazineyl)-3-oxoprop-1-en-2-y1)-3,4-dimethoxybenzamide (5a)

Yellow powder, yield: (0.30 g, 78%), m.p 243–245 °C. IR (ATR, *ν*, cm^−1^): 3421, 3376, 3246 (NH), 3052 (CH aromatic), 2948 (CH aliphatic), 1699, 1654, 1618 (C=O), 1580, 1509 (C=C), 1268, 1107 (C–O). ^1^H-NMR (400 MHz, DMSO-*d*_6_) *δ*: 10.33 (s, 2H, 2NH), 9.89 (s, 1H, NH), 8.42–8.34 (m, 1H), 7.92–7.88 (m, 1H), 7.65 (d, *J* = 7.3 Hz, 1H), 7.61 (d, *J* = 8.8 Hz, 2H), 7.54 (dt, *J* = 9.2, 4.0 Hz, 4H), 7.43 (ddd, *J* = 13.8, 7.2, 3.0 Hz, 3H), 7.23 (s, 1H, olefinic CH), 7.12–7.05 (m, 1H), 6.99 (dd, *J* = 7.8, 3.6 Hz, 1H), 4.85 (d, *J* = 8.1 Hz, 2H, OCH_2_), 3.84 (s, 3H, OCH_3_), 3.83 (s, 3H, OCH_3_). Elemental analysis for C_30_H_26_ClN_3_O_6_ (560.00): C‚ 64.34; H‚ 4.68; N‚ 7.50. Found: C‚ 64.18; H‚ 4.81; N‚ 7.58.

##### 4.1.1.4. (*Z*)-3,4-Dimethoxy-*N*-(3-(2-(2-(naphthalen-1-yloxy)acetyl)hydraziney1)-3-oxo-1-(*p*-tolyl)prop-1-en-2-yl)benzamide (5b)

Light yellow powder, yield: (0.26 g, 69%), m.p 251–253 °C. IR (ATR, *ν*, cm^−1^): 3350, 3324 (NH), 3058, 3040 (CH aromatic), 2907 (CH aliphatic), 1719, 1684, 1643 (C=O), 1599, 1578 (C=C), 1257, 1220 (C–O). ^1^H-NMR (400 MHz, DMSO-*d*_6_) *δ*: 10.28 (s, 2H), 9.80 (s, 1H), 8.35 (d, *J* = 7.5 Hz, 1H), 7.89 (d, *J* = 7.6 Hz, 1H), 7.66 (d, *J* = 9.2 Hz, 1H), 7.61 (s, 1H), 7.53 (s, 3H), 7.49 (d, *J* = 8.5 Hz, 2H), 7.42 (t, *J* = 8.0 Hz, 1H), 7.24 (s, 1H), 7.18 (d, *J* = 7.6 Hz, 2H), 7.08 (d, *J* = 9.2 Hz, 1H), 6.99 (d, *J* = 7.5 Hz, 1H), 4.83 (s, 2H), 3.84 (s, 3H), 3.83 (s, 3H), 2.29 (s, 3H). Elemental analysis for C_31_H_29_N_3_O_6_ (539.59): C‚ 69.00; H‚ 5.42; N‚ 7.79. Found: C‚ 69.13; H‚ 5.29; N‚ 7.67.

##### 4.1.1.5. (*Z*)-3,4-Dimethoxy-*N*-(1-(4-methoxypheny1)-3-(2-(2-(naphthalen-lyloxy)acetyl)hydraziney1)-3-oxoprop-1-en-2-yl)benzamide (5c)

Light yellow powder, yield: (0.28 g, 72%), m.p 233–235 °C. IR (ATR, *ν*, cm^−1^): 3395, 3279, 3212 (NH), 3059, 3003 (CH aromatic), 2967 (CH aliphatic), 1712, 1681, 1646 (C=O), 1582, 1528 (C=C), 1255, 1124 (C–O). ^1^H-NMR (400 MHz, DMSO-*d*_6_) *δ*: 10.30 (s, 2H), 9.79 (s, 1H), 8.45–8.33 (m, 1H), 8.33–8.22 (m, 1H), 7.89 (dq, *J* = 6.3, 2.8 Hz, 1H), 7.74–7.65 (m, 1H), 7.63 (s, 1H), 7.57 (dd, *J* = 8.9, 3.2 Hz, 2H), 7.54–7.49 (m, 2H), 7.43 (td, *J* = 7.9, 3.5 Hz, 1H), 7.27 (d, *J* = 4.9 Hz, 1H), 7.12 (dd, *J* = 8.3, 4.2 Hz, 1H), 7.09 (d, *J* = 8.6 Hz, 1H), 6.99 (dd, *J* = 7.6, 3.1 Hz, 1H), 6.94 (d, *J* = 8.8 Hz, 1H), 4.84 (d, *J* = 11.7 Hz, 2H), 3.96–3.87 (m, 3H), 3.84 (d, *J* = 4.7 Hz, 3H), 3.81–3.72 (m, 3H). ^13^C-NMR (101 MHz, DMSO) *δ* 166.91, 162.13, 160.18, 153.86, 148.63, 134.80, 134.49, 131.71, 127.83, 126.99, 126.48, 125.75, 125.30, 122.51, 121.86, 121.11, 115.14, 114.52, 112.40, 111.82, 111.35, 110.39, 106.15, 67.03, 56.14, 56.07, 55.68. Elemental analysis for C_31_H_29_N_3_O_7_ (555.59): C‚ 67.02; H‚ 5.26; N‚ 7.56. Found: C‚ 66.91; H‚ 5.17; N‚ 7.68.

##### 4.1.1.6. (*Z*)-*N*-(1-(4-Fluoropheny1)-3-(2-(2-(naphthalen-1-yloxy)acely1)hydraziney1)-3-oxoprop-1-en-2-y1)-3,4,5-trimethoxybenzamide (5d)

Light yellow powder, yield: (0.28 g, 70%), m.p 239–241 °C. IR (ATR, *ν*, cm^−1^): 3331, 3260 (NH), 3053 (CH aromatic), 2957 (CH aliphatic), 1699, 1660, 1638 (C=O), 1580, 1512 (C=C), 1259, 1177 (C–O). ^1^H-NMR (400 MHz, DMSO-*d*_6_) *δ*: 10.30 (s, 1H), 9.95 (s, 1H), 9.45 (s, 1H), 8.36 (dd, *J* = 7.4, 2.8 Hz, 1H), 7.89 (ddd, *J* = 8.0, 5.1, 2.7 Hz, 2H), 7.67 (dd, *J* = 8.4, 5.7 Hz, 1H), 7.55–7.52 (m, 3H), 7.44–7.41 (m, 1H), 7.37 (s, 2H), 7.28 (d, *J* = 5.5 Hz, 1H), 7.24 (d, *J* = 8.7 Hz, 1H), 7.00 (d, *J* = 7.7 Hz, 1H), 6.92 (d, *J* = 7.6 Hz, 1H), 4.84 (s, 2H), 3.86 (s, 6H), 3.74 (s, 3H). ^13^C-NMR (101 MHz, DMSO) *δ*: 167.07, 166.95, 165.83, 164.77, 153.83, 153.02, 140.89, 134.50, 132.10, 129.11, 127.83, 127.00, 126.47, 125.73, 125.29, 122.64, 121.07, 116.19, 115.97, 106.21, 106.16, 106.09, 105.94, 67.17, 60.58, 56.54. Elemental analysis for C_31_H_28_FN_3_O_7_ (573.58): C‚ 64.92; H‚ 4.92; N‚ 7.33. Found: C‚ 65.04; H‚ 5.02; N‚ 7.23.

##### 4.1.1.7. (*Z*)-*N*-(1-(4-Chloropheny1)-3-(2-(2-(naphthelen-1-yloxy)ecetyl)hydraziney1)-3-oxoprop-1-en-2-y1)-3,4,5-trimethorybeneamide (5e)

Yellow powder, yield: (0.31 g, 75%), m.p 235–237 °C. IR (ATR, *ν*, cm^−1^): 3349, 3280 (NH), 3057, 3007 (CH aromatic), 2963, 2935 (CH aliphatic), 1709, 1678, 1629 (C=O), 1582, 1510 (C=C), 1257, 1132 (C–O). ^1^H-NMR (400 MHz, DMSO-*d*_6_) *δ*: 10.33 (s, 1H), 10.22 (s, 1H), 9.92 (s, 1H), 8.40–8.32 (m, 1H), 7.89 (dd, *J* = 6.8, 2.6 Hz, 1H), 7.57–7.49 (m, 5H), 7.44 (d, *J* = 7.9 Hz, 1H), 7.38 (s, 2H), 7.28 (s, 1H), 7.20 (d, *J* = 8.0 Hz, 2H), 7.00 (d, *J* = 7.6 Hz, 1H), 4.84 (s, 2H), 3.86 (s, 6H), 3.75 (s, 3H). ^13^C-NMR (101 MHz, DMSO) *δ* 167.10, 166.38, 165.82, 164.97, 153.85, 153.01, 140.84, 139.18, 134.50, 131.59, 130.55, 130.17, 130.01, 129.68, 129.25, 128.43, 127.83, 127.00, 126.48, 125.76, 125.31, 122.52, 121.15, 106.16, 106.09, 66.87, 60.58, 56.53. Elemental analysis for C_31_H_28_ClN_3_O_7_ (590.03): C‚ 63.11; H‚ 4.78; N‚ 7.12. Found: C‚ 62.97; H‚ 4.87; N‚ 7.25.

##### 4.1.1.8. (*Z*)-3,4,5-Trimethoxy-*N*-(3-(2-(2-(naphthalen-1-yloxy)acetyl)hydraziney1)-3-ozo-1-(*p*-tolyl)prop-1-en-2-yl)benzamide (5f)

Yellow powder, yield: (0.27 g, 68%), m.p 223–225 °C. IR (ATR, *ν*, cm^−1^): 3455, 3411, 3228 (NH), 3056, 3005 (CH aromatic), 2937, 2836 (CH aliphatic), 1698, 1659, 1628 (C=O), 1582, 1548 (C=C), 1255, 1124 (C–O). ^1^H-NMR (400 MHz, DMSO-*d*_6_) *δ*: 10.38 (s, 1H), 10.32 (s, 1H), 9.99 (s, 1H), 8.37 (d, *J* = 7.5 Hz, 1H), 7.89 (d, *J* = 7.3 Hz, 1H), 7.68–7.59 (m, 3H), 7.55 (dd, *J* = 10.4, 6.2 Hz, 4H), 7.44 (d, *J* = 7.8 Hz, 1H), 7.38 (s, 2H), 7.25 (s, 1H), 7.00 (d, *J* = 7.6 Hz, 1H), 4.86 (s, 2H), 3.87 (s, 6H), 3.75 (s, 3H), 2.51 (s, 3H). ^13^C-NMR (101 MHz, DMSO) *δ* 172.49, 167.12, 165.82, 164.78, 153.85, 153.04, 140.96, 134.51, 133.76, 132.06, 131.81, 130.07, 129.03, 128.82, 127.83, 127.01, 126.47, 125.77, 125.32, 122.52, 121.16, 106.15, 106.13, 66.87, 60.58, 56.55, 19.03. Elemental analysis for C_32_H_31_N_3_O_7_ (569.61): C‚ 67.48; H‚ 5.49; N‚ 7.38. Found: C‚ 67.37; H‚ 5.61; N‚ 7.47.

##### 4.1.1.9. (*Z*)-3,4,5-Trimethoxy-*N*-(1-(4-methoxypheny1)-3-(2-(2-(naphthalen-1-yloxy)acetyl)hydrazineyl)-3-oxoprop-1-en-2-yl)benzamide (5g)

Yellow powder, yield: (0.32 g, 79%), m.p 217–219 °C. IR (ATR, *ν*, cm^−1^): 3455, 3357, 3246 (NH), 3052 (CH aromatic), 2969 (CH aliphatic), 1769, 1700, 1658 (C=O), 1579, 1507 (C=C), 1237, 1178 (C–O). ^1^H-NMR (400 MHz, DMSO-*d*_6_) *δ*: 10.30 (s, 2H), 9.89 (s, 1H), 8.43–8.33 (m, 1H), 7.94–7.85 (m, 1H), 7.61 (dd, *J* = 18.8, 8.6 Hz, 2H), 7.53 (dq, *J* = 9.5, 5.5, 4.4 Hz, 3H), 7.46–7.41 (m, 1H), 7.39 (s, 2H), 7.29 (s, 1H), 7.05–6.97 (m, 2H), 6.96 (s, 1H), 4.85 (d, *J* = 10.0 Hz, 2H), 3.86 (s, 6H), 3.77 (s, 3H), 3.75 (s, 3H). ^13^C-NMR (101 MHz, DMSO) *δ*: 167.06, 165.79, 165.04, 160.27, 153.85, 153.01, 140.81, 134.50, 131.77, 129.30, 127.83, 127.00, 126.85, 126.47, 125.76, 125.30, 122.60, 122.51, 121.14, 114.59, 106.21, 106.16, 106.09, 66.89, 60.58, 56.54, 55.70. Elemental analysis for C_32_H_31_N_3_O_8_ (585.61): C‚ 65.63; H‚ 5.34; N‚ 7.18. Found: C‚ 65.78; H‚ 5.22; N‚ 7.26.

##### 4.1.1.10. (*Z*)-*N*-(1-(3,4-Dimethoxypheny1)-3-(2-(2-(naphthalen-1-yloxy)acetyl) hydraziney1)-3-oxoprop-1-en-2-y1)-3,4,5-trimethoxybenzamide (5h)

Orange powder, yield: (0.30 g, 71%), m.p 229–231 °C. IR (ATR, *ν*, cm^−1^): 3366, 3256 (NH), 3053, 3002 (CH aromatic), 2938 (CH aliphatic), 1690, 1656, 1623 (C=O), 1594, 1495 (C=C), 1278, 1095 (C–O). ^1^H-NMR (400 MHz, DMSO-*d*_6_) *δ*: 10.32 (s, 1H), 10.21 (s, 1H), 9.83 (s, 1H), 8.40–8.31 (m, 1H), 7.93–7.84 (m, 1H), 7.72–7.65 (m, 2H), 7.56–7.49 (m, 3H), 7.41 (t, *J* = 7.9 Hz, 1H), 7.32 (s, 1H), 7.11–7.02 (m, 2H), 6.99 (d, *J* = 3.8 Hz, 2H), 4.83 (s, 2H), 3.83 (s, 3H), 3.81 (s, 3H), 3.65 (s, 3H), 3.61 (s, 6H). ^13^C-NMR (101 MHz, DMSO) *δ* 167.00, 165.90, 164.85, 153.84, 153.04, 152.14, 148.57, 138.58, 134.49, 131.10, 129.78, 128.47, 127.83, 127.00, 126.47, 126.31, 125.75, 125.30, 122.52, 121.94, 121.14, 111.70, 111.23, 107.65, 106.16, 66.88, 60.52, 56.12, 56.10, 56.07. Elemental analysis for C_33_H_33_N_3_O_9_ (615.64): C‚ 64.38; H‚ 5.40; N‚ 6.83. Found: C‚ 64.29; H‚ 5.31; N‚ 7.03.

##### 4.1.1.11. (*Z*)-3,4,5-Trimethoxy-*N*-(3-(2-(2-(naphthalen-1-yloxy)acetyl)hydraziney1)-3-oxo-1-(3,4,5-trimethoxyphenyl)prop-1-en-2-yl)benzamide (5i)

Orange powder, yield: (0.30 g, 68%), m.p 211–213 °C. IR (ATR, *ν*, cm^−1^): 3455, 3364, 3250 (NH), 3060, 3005 (CH aromatic), 2964, 2939 (CH aliphatic), 1698, 1650, 1630 (C=O), 1587, 1496 (C=C), 1270, 1180 (C–O). ^1^H-NMR (400 MHz, DMSO-*d*_6_) *δ*: 10.32 (s, -1H), 8.44–8.35 (m, 1H), 7.91 (s, 2H), 7.88 (s, 1H), 7.84 (s, 2H), 7.57–7.52 (m, 3H), 7.44 (d, *J* = 7.9 Hz, 1H), 7.12 (s, 1H), 6.99 (d, *J* = 7.6 Hz, 1H), 4.86 (s, 2H), 3.87 (s, 6H), 3.85 (s, 6H), 3.78 (s, 3H), 3.75 (s, 3H). ^13^C NMR (101 MHz, DMSO) *δ* 170.63, 168.68, 167.11, 159.74, 153.73, 153.19, 152.93, 141.27, 139.99, 136.66, 134.50, 130.26, 127.83, 127.04, 126.47, 125.78, 125.26, 123.94, 122.59, 121.26, 110.29, 107.40, 106.21, 66.97, 60.73, 60.68, 56.30, 56.10. Elemental analysis for C_34_H_35_N_3_O_10_ (645.67): C‚ 63.25; H‚ 5.46; N‚ 6.51. Found: C‚ 63.38; H‚ 5.58; N‚ 6.40.

### 4.2. Biological evaluation

The experimental details for the performed biological profiling of the target naphthalene–enamide analogs 4a,b and 5a–i reported in this work were explained in the SI.

## Conflicts of interest

No potential conflict of interest was reported by the author (s).

## Supplementary Material

RA-015-D5RA07730C-s001

## Data Availability

The authors confirm that the data supporting the findings of this study are available within the article and/or its supporting information (SI). Supplementary information is available. See DOI: https://doi.org/10.1039/d5ra07730c.
