# Supplementary material for: New naphthalene-containing enamides: synthesis, structural insights and biological screening as potential anticancer agents against Huh-7 cancer cell line
Source: RSC Adv. 2025 Dec 16;15(58):50166–78. doi: 10.1039/d5ra07730c (PMC12707369; doi:10.1039/d5ra07730c)
Supplement: RA-015-D5RA07730C-s001 [file RA-015-D5RA07730C-s001.pdf]

Supplementary Information (SI) for RSC Advances.
This journal is © The Royal Society of Chemistry 2025

New Naphthalene-Containing Enamides: Synthesis, Structural Insights and Biological Screening as Potential Anticancer Agents against Huh-7 Cancer Cell Line

Rasha Mohammed Saleem [1], Arwa Sultan Alqahtani [2], Rabah N. Alsulami [3] and Islam Zaki [4,*]

[1] Department of Laboratory Medicine, Faculty of Applied Medical Sciences, Al-Baha University, Al-Baha, 65431, Saudi Arabia; [2] Department of Chemistry, College of Science, Imam Mohammad Ibn Saud Islamic University (IMSIU), P.O. Box, 90950, Riyadh 11623, Saudi Arabia; [3] Department of Biology, College of Applied Sciences, Umm Al-Qura University, Saudi Arabia; [4] Pharmaceutical Organic Chemistry Department, Faculty of pharmacy, Port Said University, Port Said, Egypt

---

** To whome correspondence should be addressed*

Islam Zaki, PhD. Pharmaceutical Organic Chemistry Department, Faculty of pharmacy, Port Said University, Port Said, Egypt.

E-mail address: Eslam.Zaki@pharm.psu.edu.eg **(I. Zaki)**

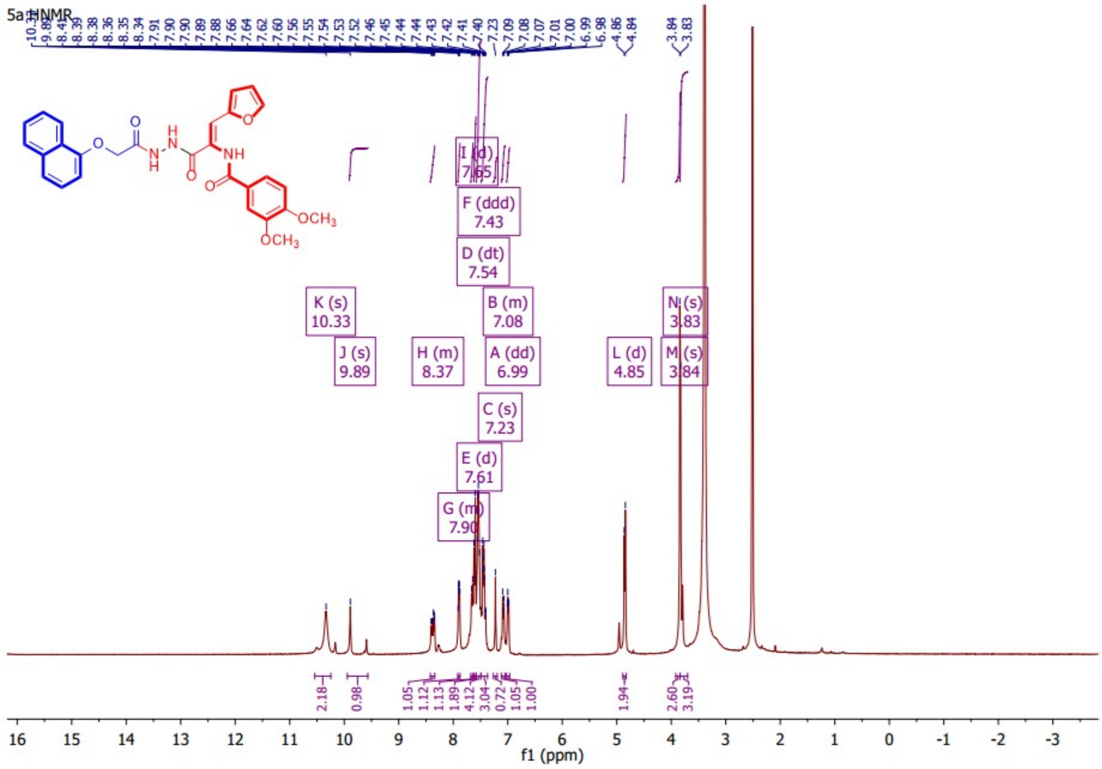

Figure S1: ^{1}H-NMR spectrum of compound **4a** in DMSO-*d6*

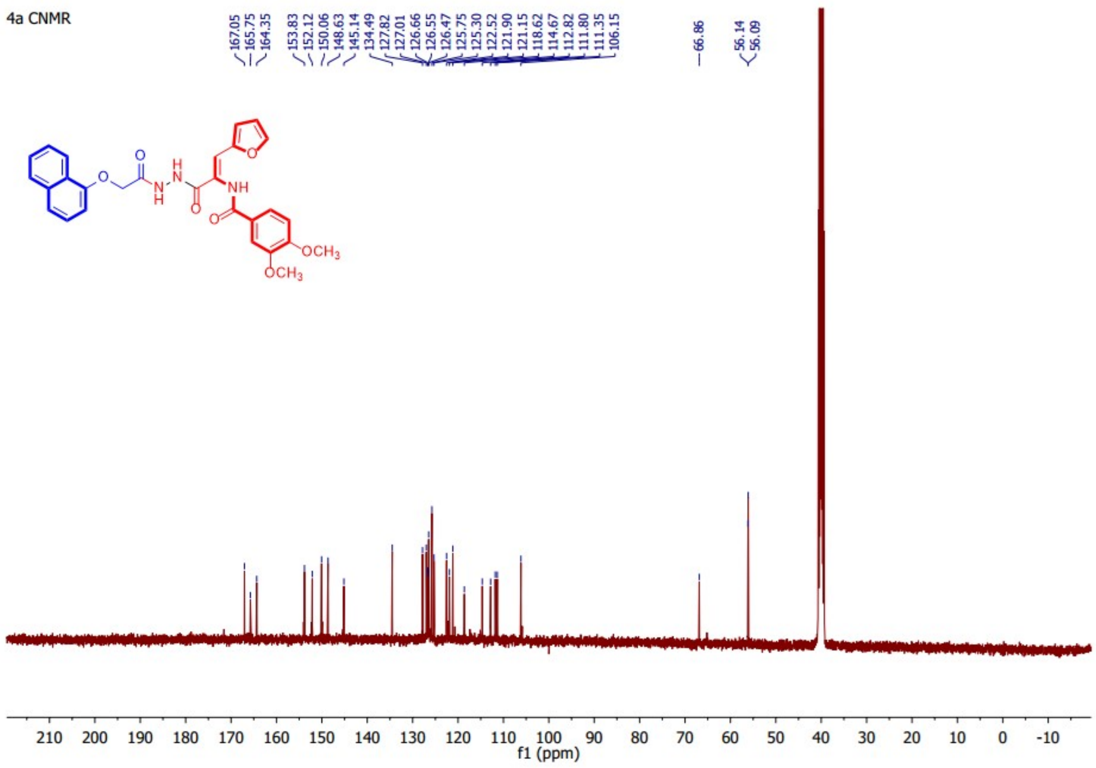

Figure S2: ^{13}C-NMR spectrum of compound **4a** in DMSO-*d6*

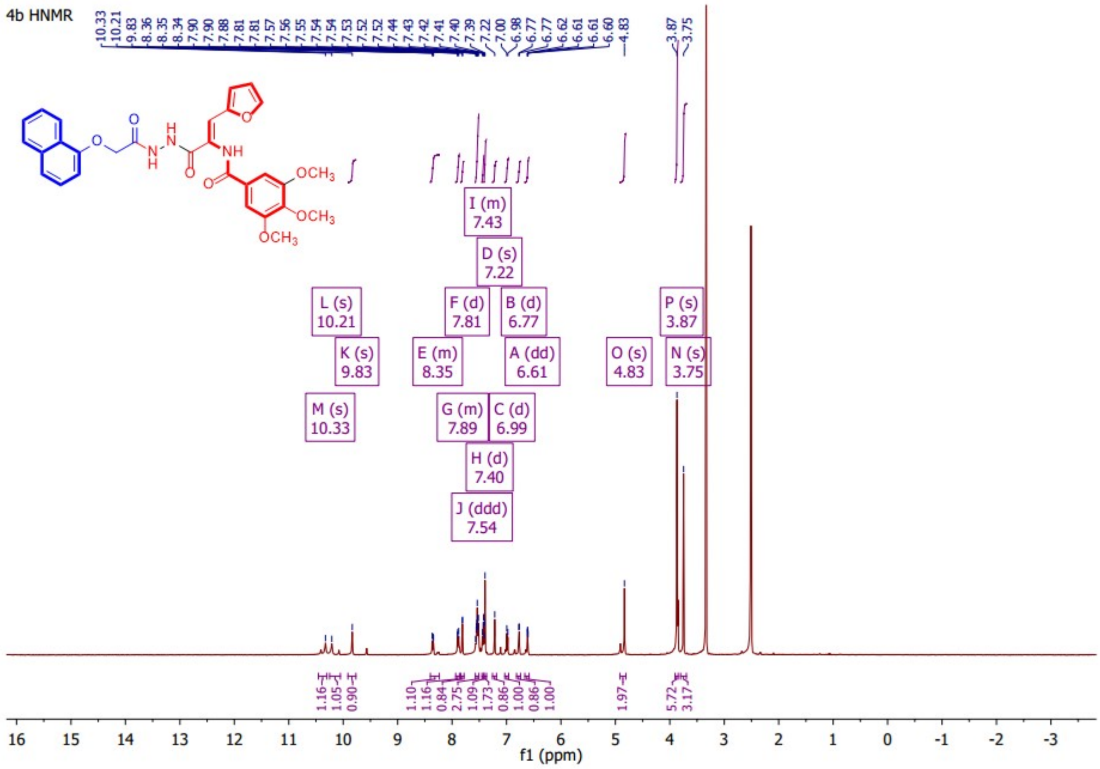

Figure S3: ^{1}H-NMR spectrum of compound **4b** in DMSO-*d6*

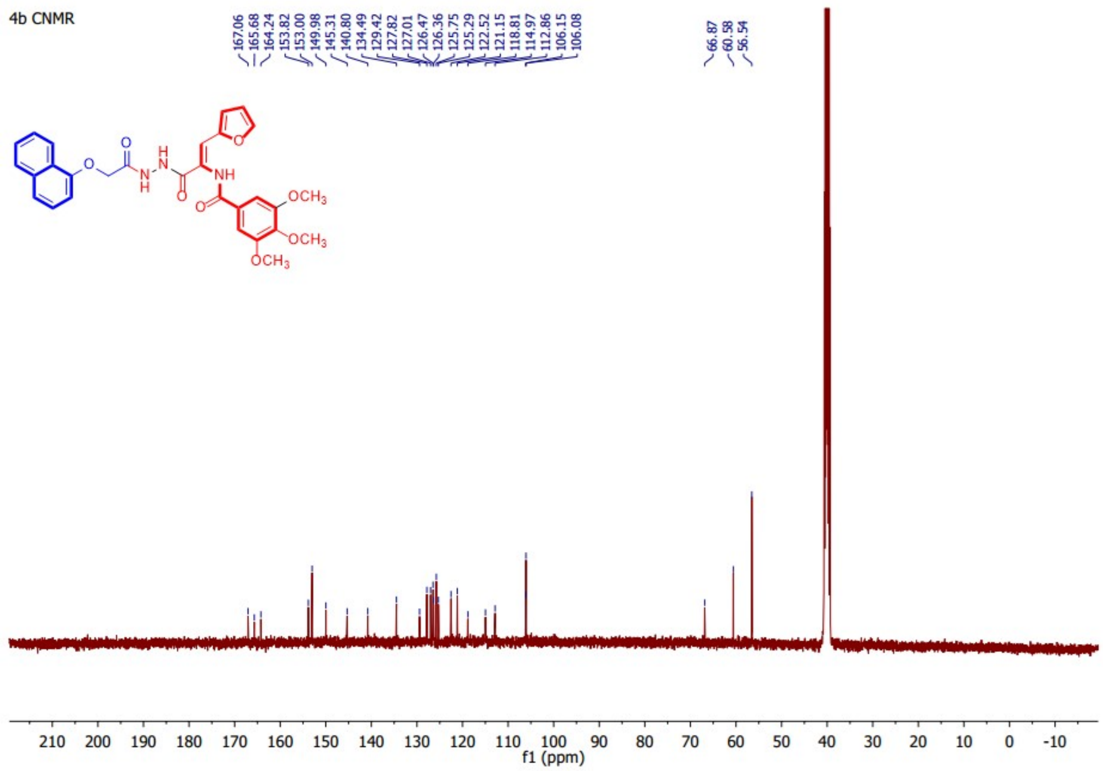

Figure S4: ^{13}C-NMR spectrum of compound **4b** in DMSO-*d6*

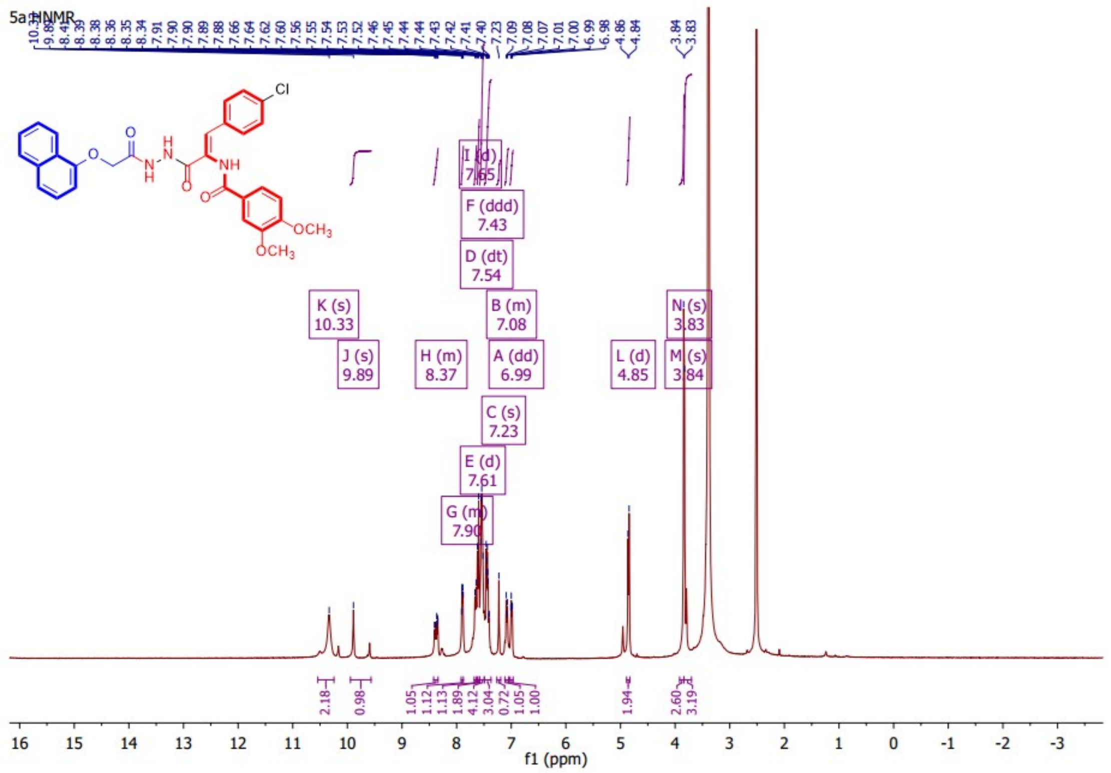

Figure S5: ^{1}H-NMR spectrum of compound **5a** in DMSO-*d6*

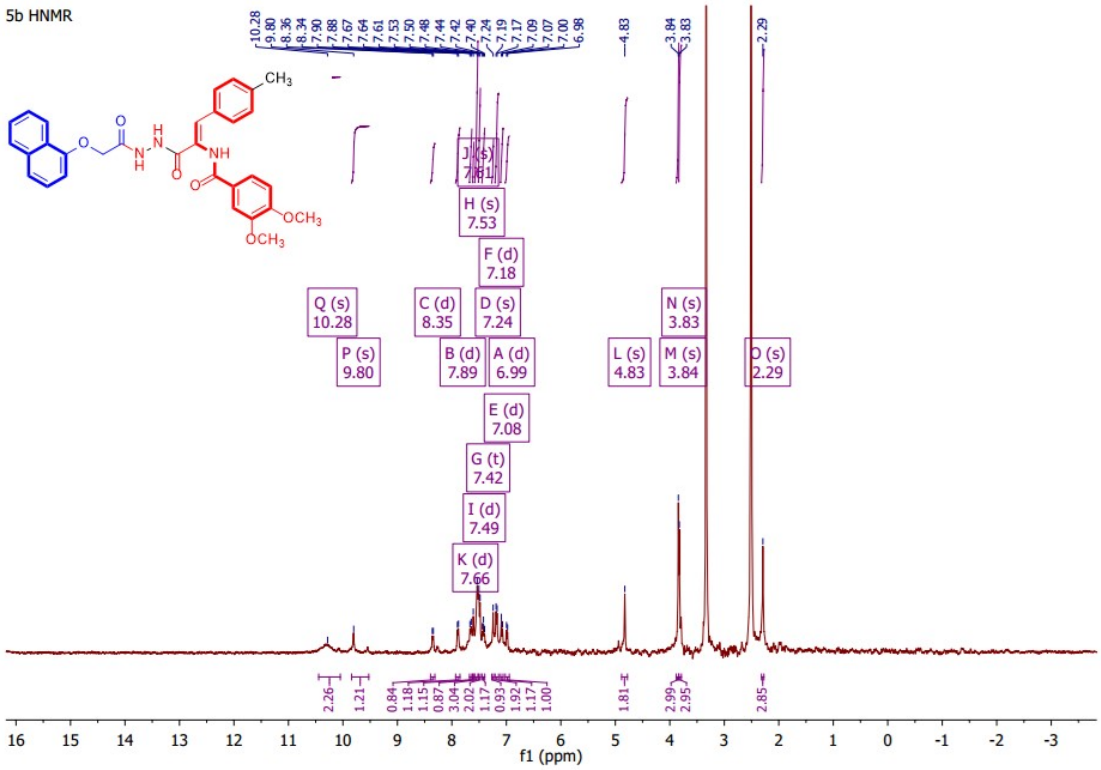

Figure S6: ^{1}H-NMR spectrum of compound **5b** in DMSO-*d6*

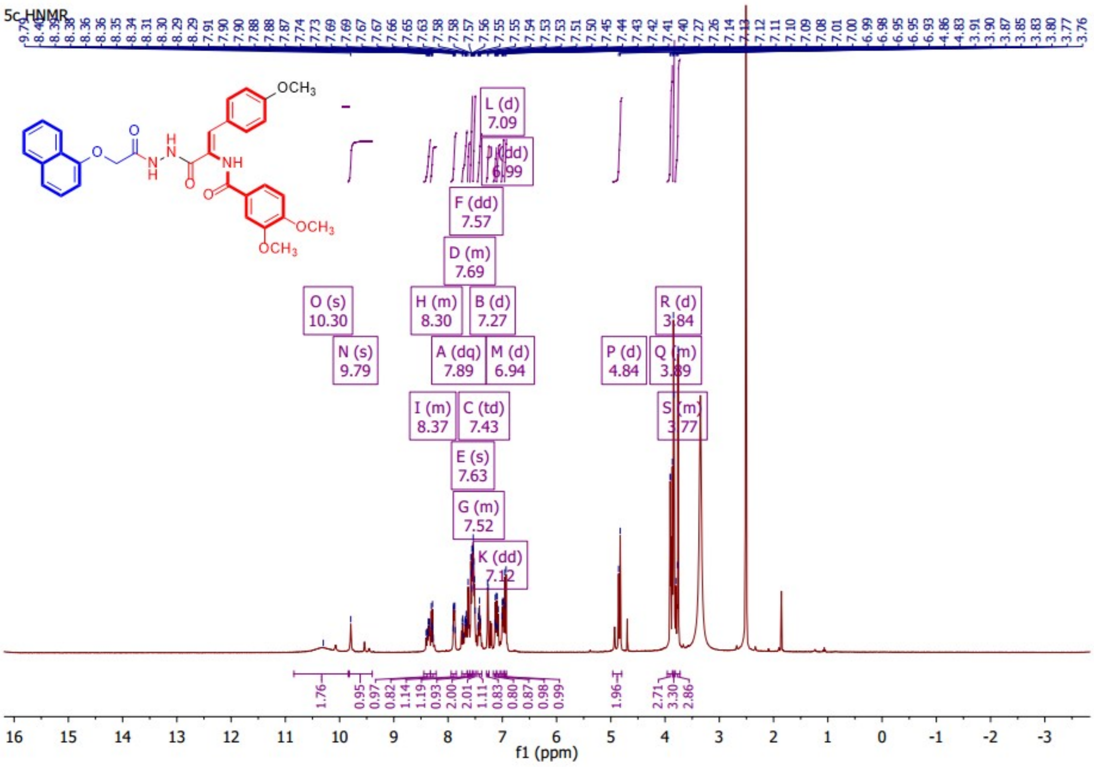

Figure S7: ^{1}H-NMR spectrum of compound **5c** in DMSO-*d6*

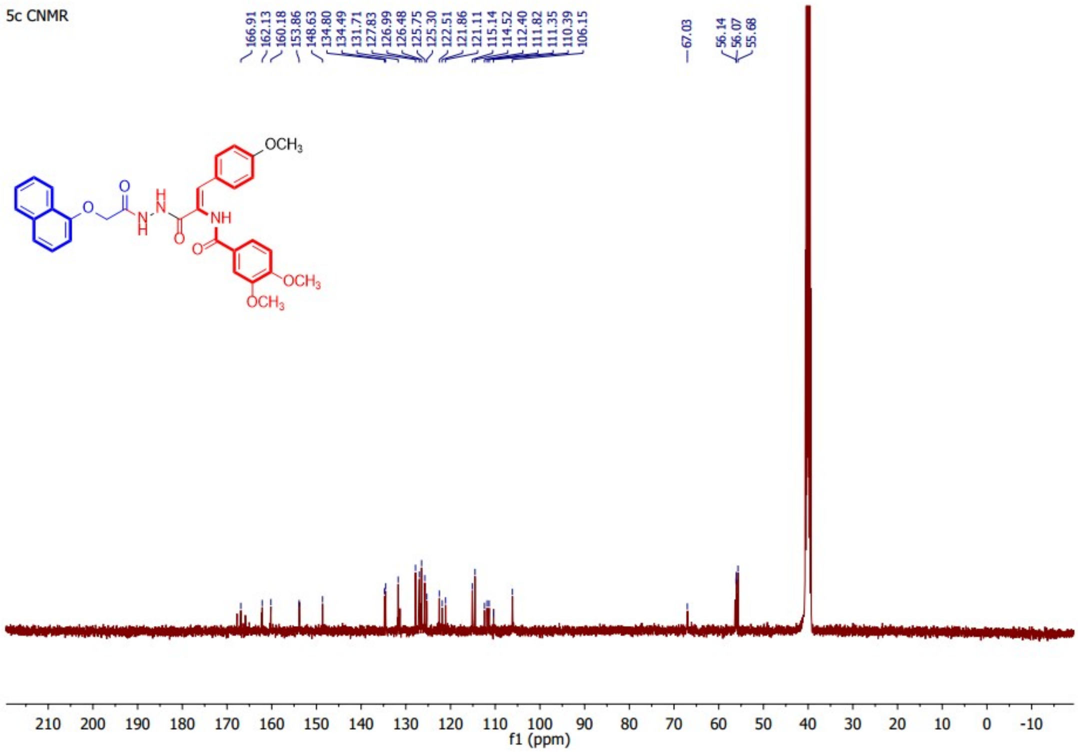

Figure S8: ^{13}C-NMR spectrum of compound **5c** in DMSO-*d6*

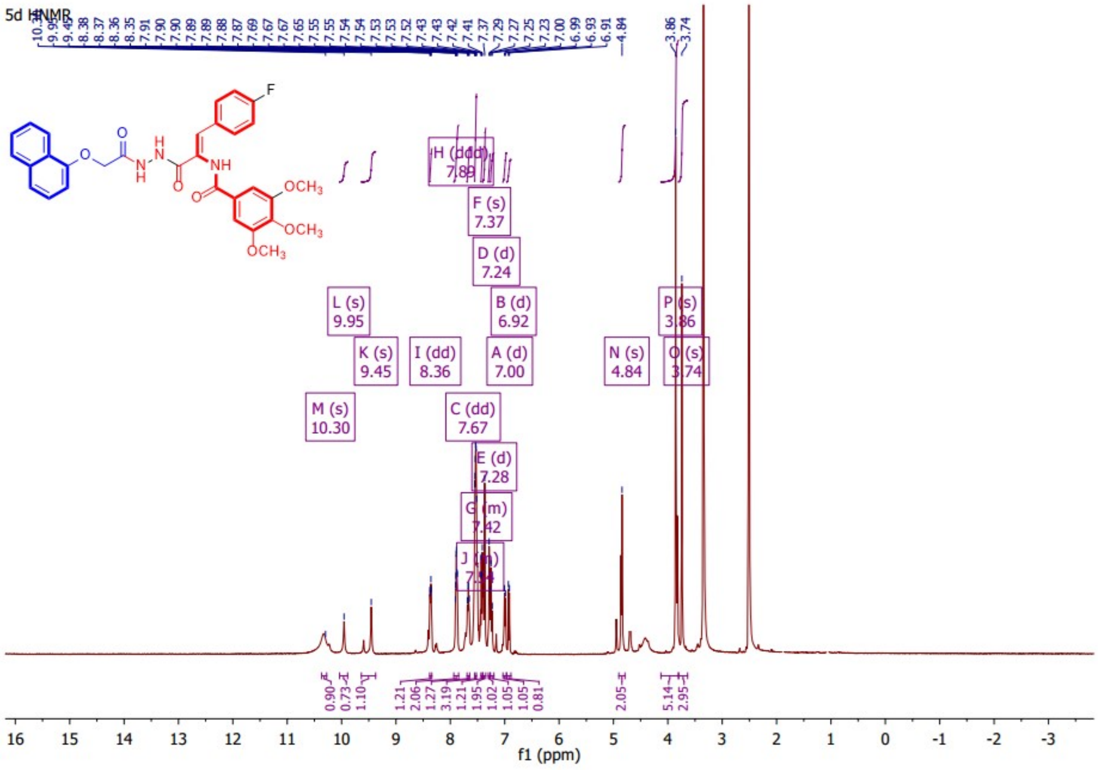

Figure S9: ^{1}H-NMR spectrum of compound **5d** in DMSO-*d6*

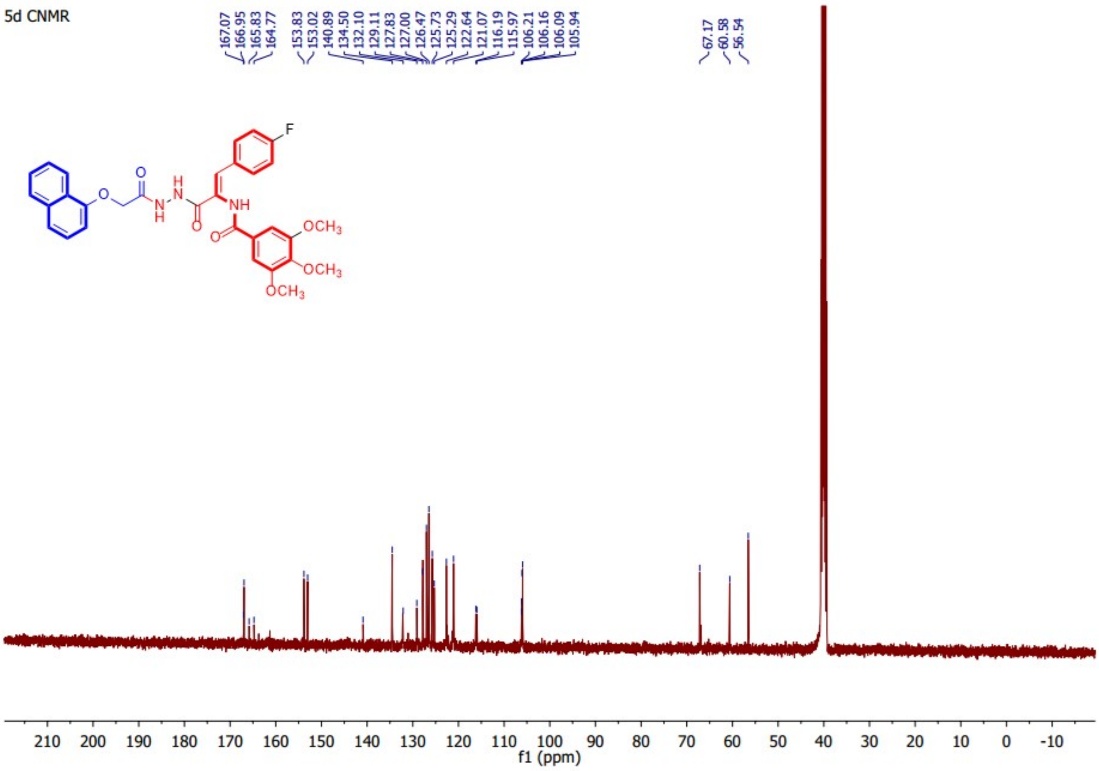

Figure S10: ^{13}C-NMR spectrum of compound **5d** in DMSO-*d6*

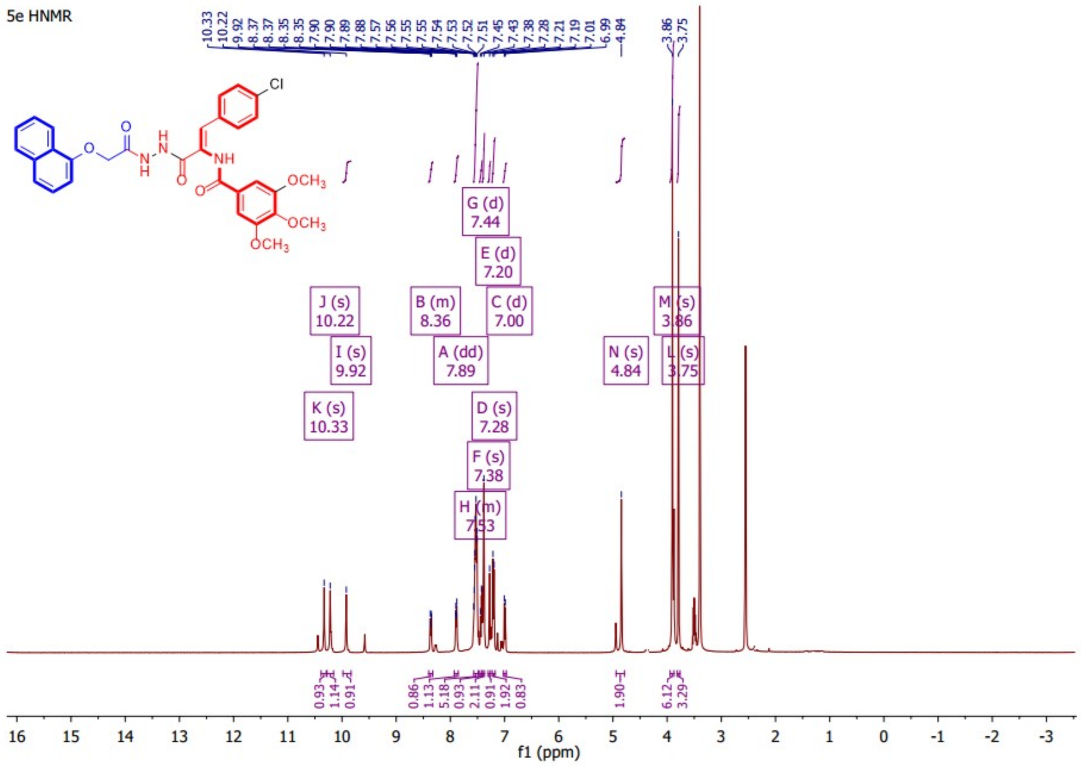

Figure S11: ^{1}H-NMR spectrum of compound **5e** in DMSO-*d6*

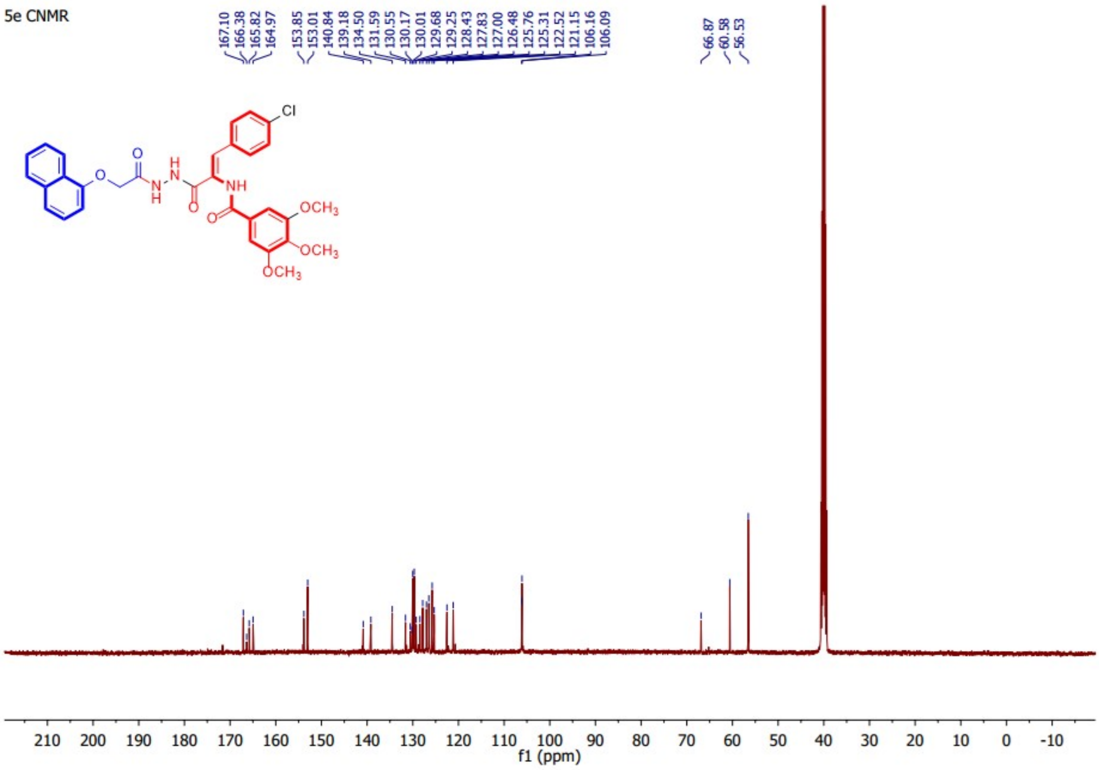

Figure S12: ^{13}C-NMR spectrum of compound **5e** in DMSO-*d6*

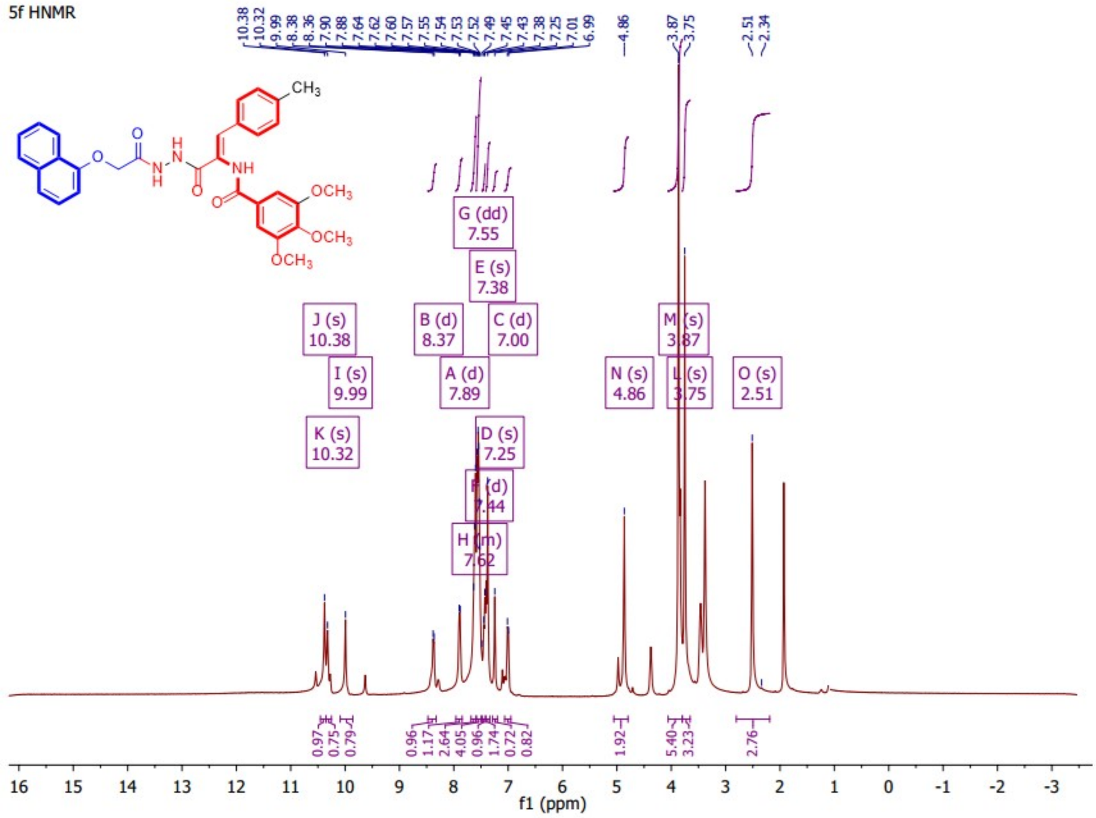

Figure S13: ^{1}H-NMR spectrum of compound **5f** in DMSO-*d6*

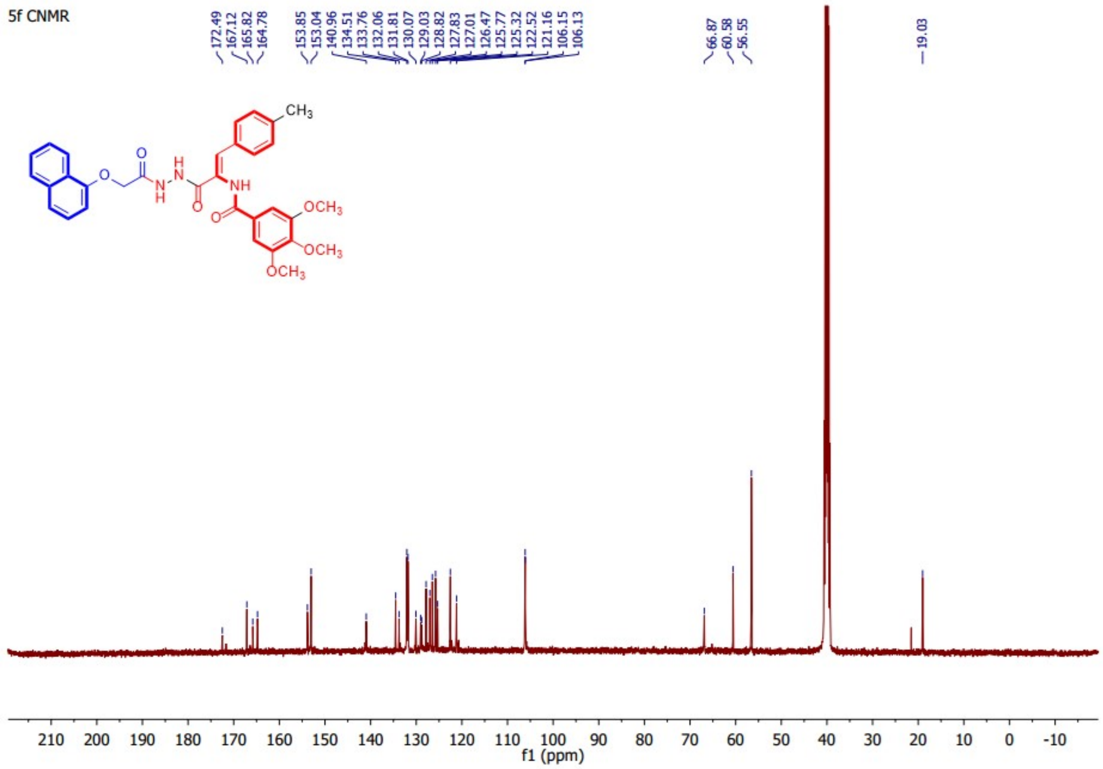

Figure S14: ^{13}C-NMR spectrum of compound **5f** in DMSO-*d6*

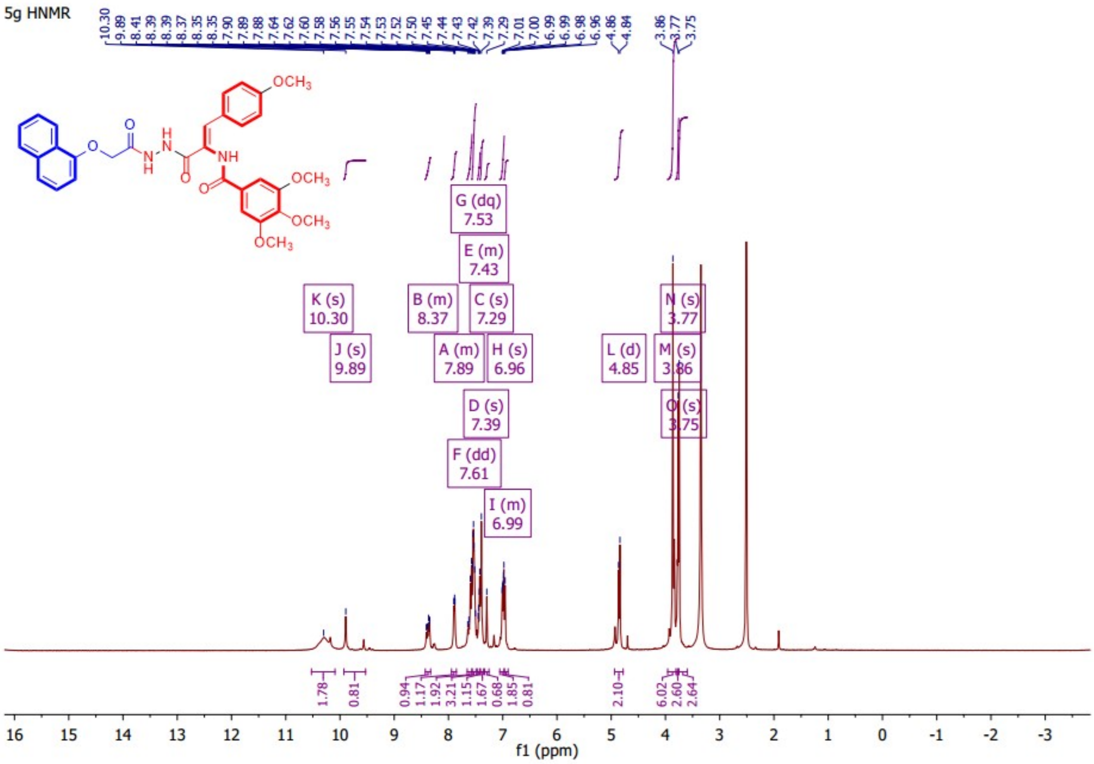

Figure S15: ^{1}H-NMR spectrum of compound **5g** in DMSO-*d6*

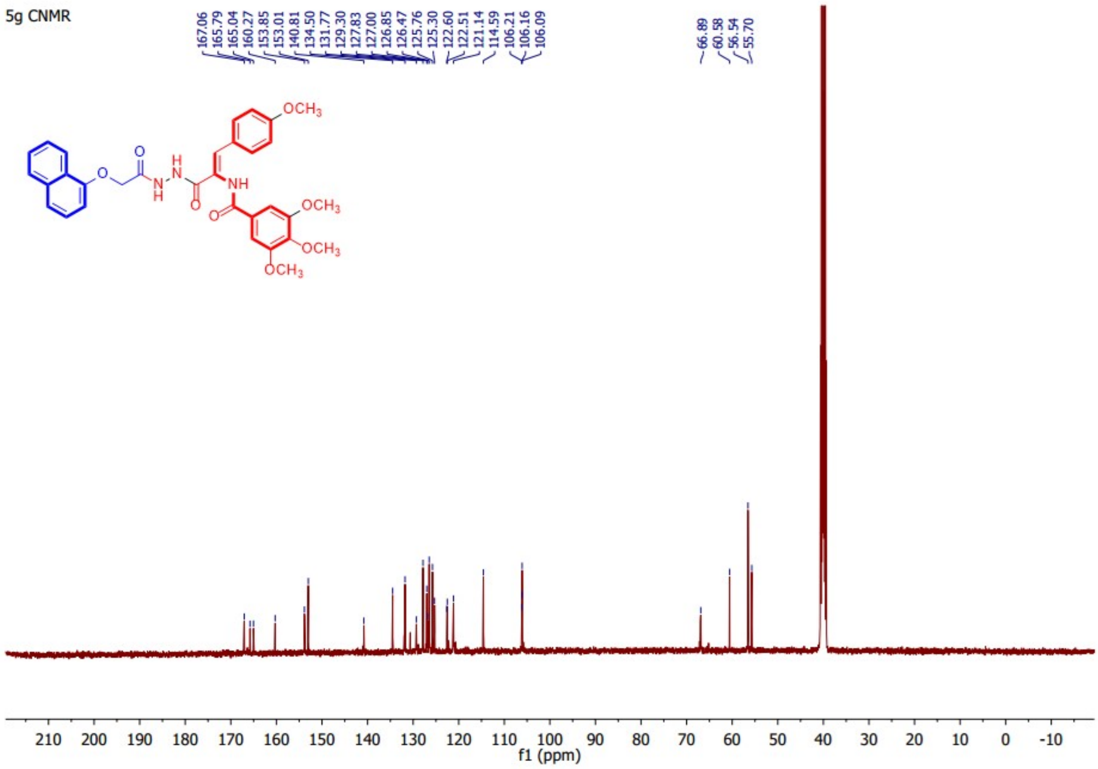

Figure S16: ^{13}C-NMR spectrum of compound **5g** in DMSO-*d6*

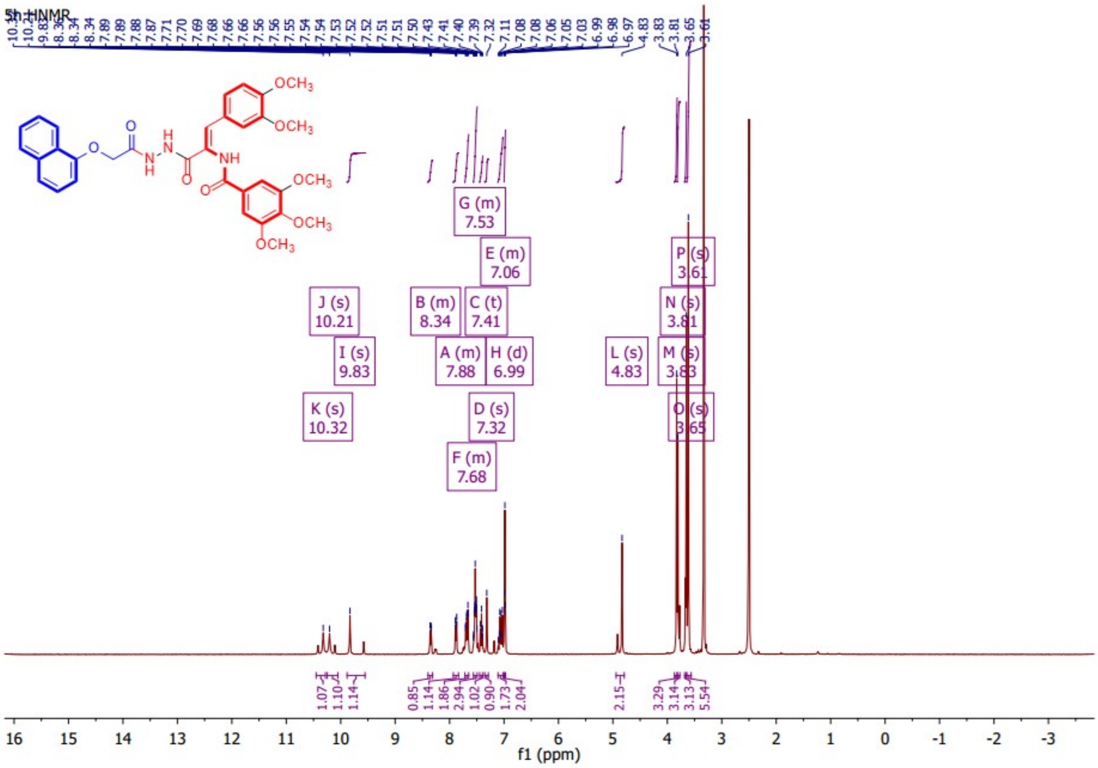

Figure S17: ^{1}H-NMR spectrum of compound **5h** in DMSO-*d6*

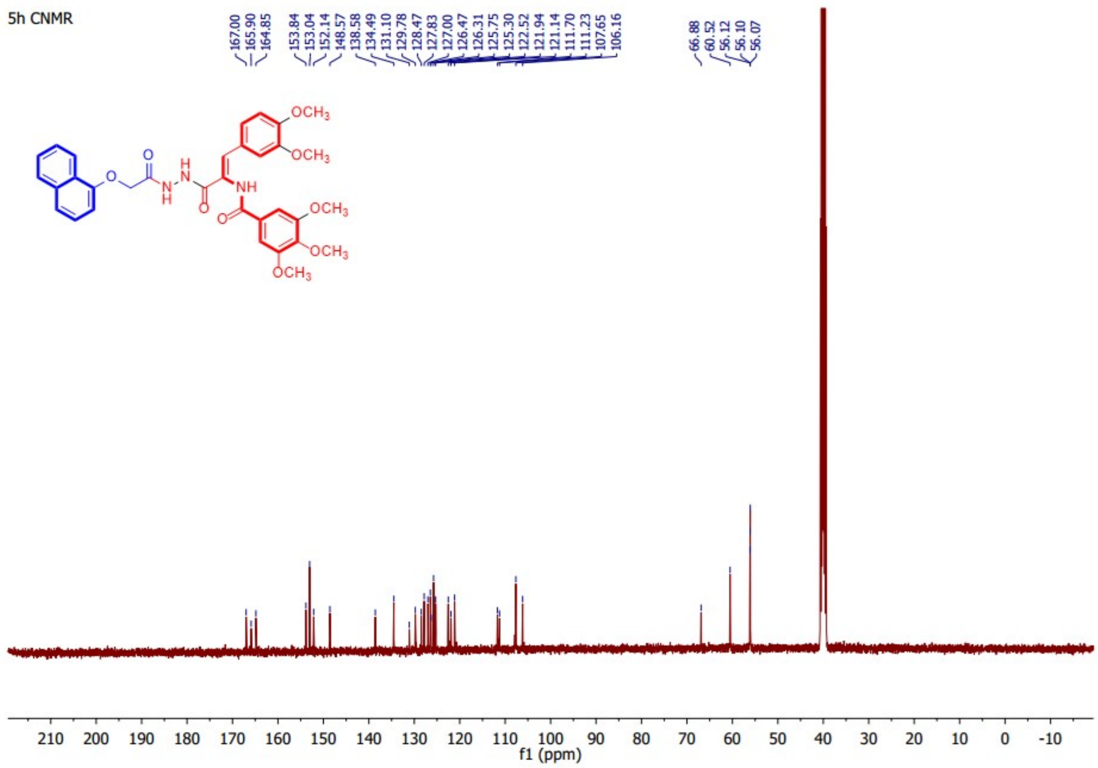

Figure S18: ^{13}C-NMR spectrum of compound **5h** in DMSO-*d6*

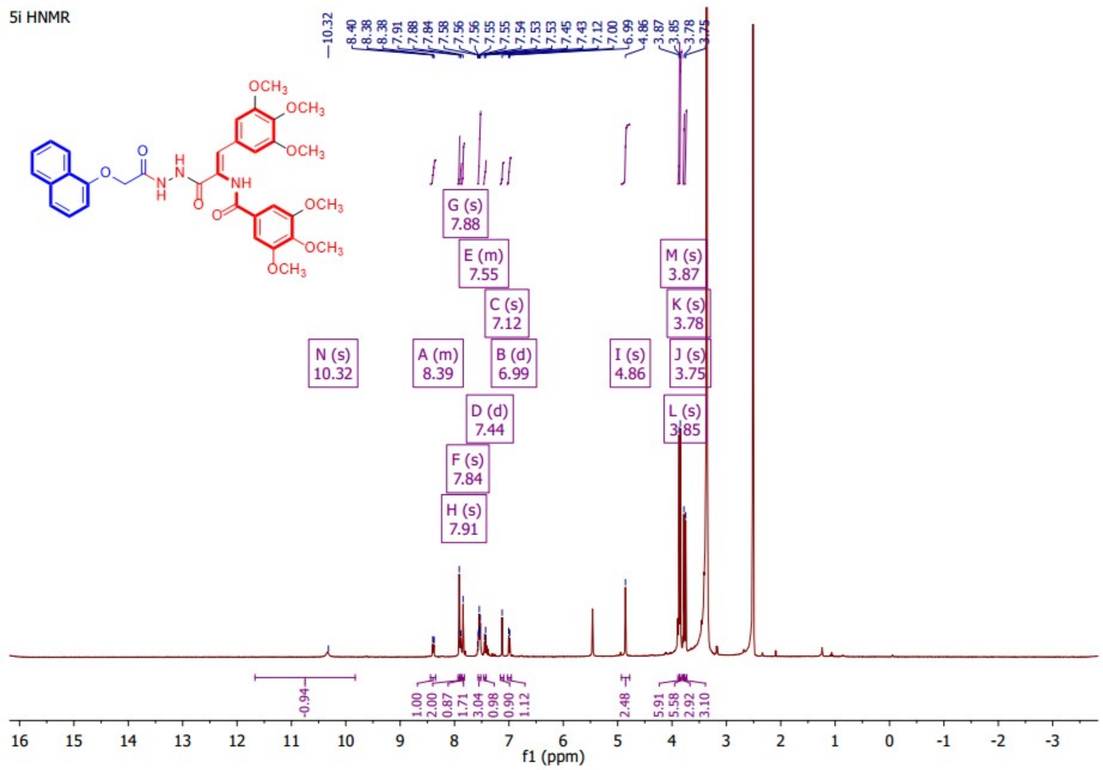

Figure S19: ^{1}H-NMR spectrum of compound **5i** in DMSO-*d6*

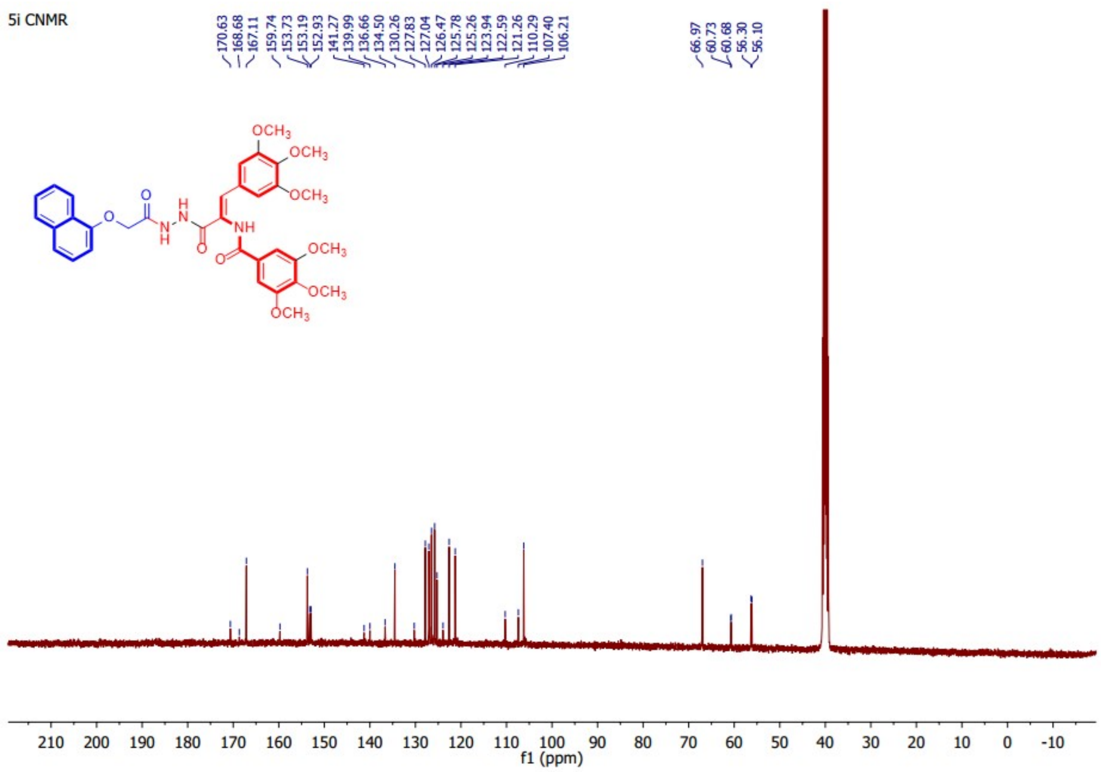

Figure S20: ^{13}C-NMR spectrum of compound **5i** in DMSO-*d6*

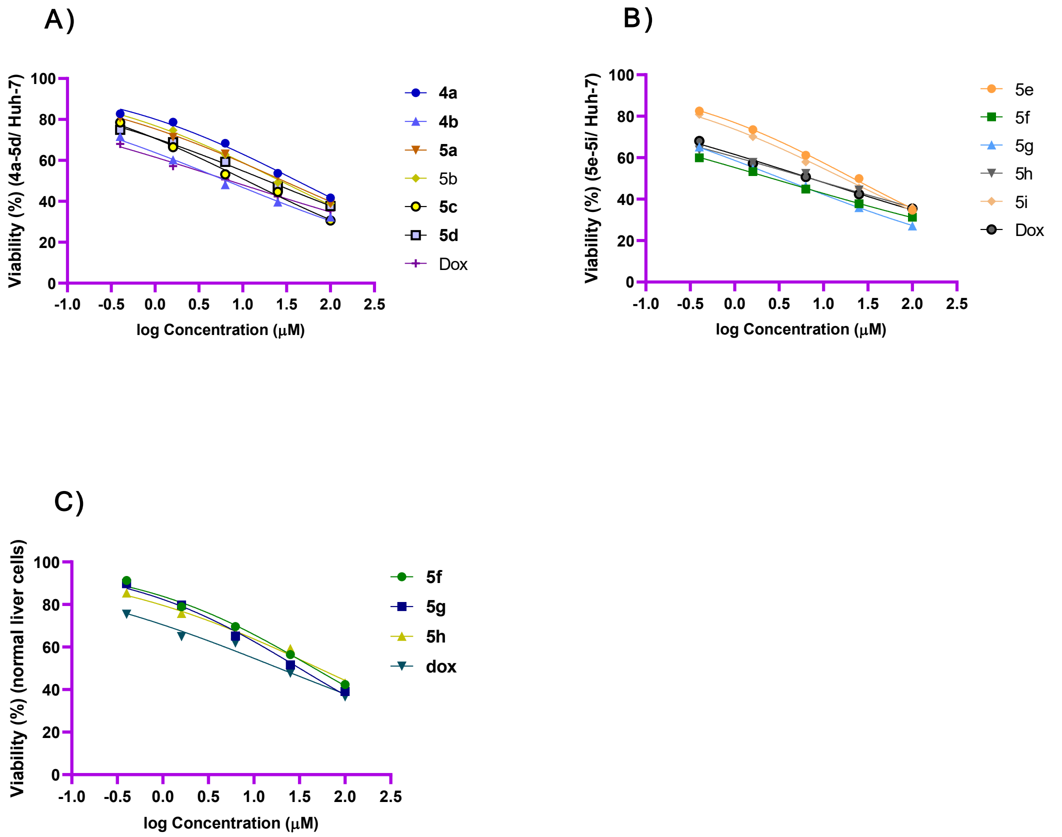

Figure S21: Dose response curve for the influence of the synthesized naphthalene-enamide compounds **4a,b** and **5a-i** at five different concentration (µM) on Huh-7 and normal liver THLE-2 cell lines for 24 h. **(A)** Influence of compounds **4a,b** and **5a-d** on Huh-7 cells. **(B)** Influence of compounds **5e-i** on Huh-7 cells. **(C)** Influence of compounds **5f**, **5g** and **5h** on THLE-2 cells. Values represent the mean ± SD for three replicates.

Appendix A

S4.2. Biological Studies

S4.2.1. Cytotoxic activity evaluation

To measure the cytotoxic actvity of the synthesized naphthalene tethered enamide derivatives **4a, b** and **5a-i** in hepatocellular carcinoma (Huh-7) cell line (JCRB Cat. No. JCRB0403) as well as normal liver (THLE-2) cell line (ATCC Cat. No. CRL-2706). Cell viability assay was assessed using MTT assay method. Cells at density of 1 x 10^4 were seeded in a 96-well plate at 37 ^oC for 24 h under 5% CO_2. After incubation, the cells were treated with different concentrations of the investigated naphthalene-tethered enamide derivatives **4a, b** and **5a-i** and incubated for 24 h, then 20 μl of MTT solution at 5 mg/mL was applied and incubated for 4 h at 37 °C. Dimethyl sulphoxide (DMSO) in volume of 100 μl was added to each well to dissolve the purple formazan that had formed. The color intensity of the formazan product, which represents the growth condition of the cells, is quantified by using an ELISA plate reader (EXL 800, USA) at 570 nm absorbance. The experimental conditions were carried out with at least three replicates, and the experiments were repeated at least three times.

S4.2.2. *In vitro* measurement of ß-tubulin inhibition percentage assay

Compounds **5f, 5g, 5h** and **Podo** were evaluated for their tubulin inhibitory activity according to manufacturer's instructions. Briefly, Huh-7 cell line was cultured using DMEM (Invitrogen/Life Technologies) supplemented with 10% FBS (Hyclone), 10 μg/mL of insulin (Sigma), and 1% penicillin-streptomycin. Plate cells in a volume of 100 μL complete growth medium and 100 μL of the tested naphthalene-enamide analogs per well in a 96-well plate for 18-24 h before the enzyme assay for Tubulin. The microtiter plate provided in this kit has been pre-coated with antibody specific to TUBß. Standards or samples are then added to the appropriate microtiter plate wells with a biotin-conjugated antibody specific to TUBb. Next, Avidin conjugated to Horseradish Peroxidase (HRP) is added to each microplate well and incubated. After TMB substrate solution is added, only those wells that contain TUBß, biotin-conjugated antibody and enzyme-conjugated Avidin will exhibit a change in colour.

The enzyme-substrate reaction is terminated by the addition of sulphuric acid solution and the colour change is measured spectrophotometrically at a wavelength of 450 nm ± 10 nm. The concentration of TUBß in the samples is then determined by comparing the O.D. of the samples to the standard curve. All experiments were done in duplicates.

S4.2.3. Cell cycle analysis of compound 5f

Cell cycle analysis in Huh-7 cells was investigated using fluorescent Annexin V-FITC/ PI detection kit (*BioVision* EZCell™ Cell Cycle Analysis Kit Catalog #K920) by flow cytometry assay. Huh-7 cells at a density of 2×10^5 per well were harvested and washed twice in PBS. After that, the cells were incubated at 37 °C and 5% CO_2. The medium was incubated with the tested compound **5f** at the IC_{50} (μM) for 48 h, washed twice in PBS, fixed with 70% ethanol, rinsed again with PBS. Afterward, medium was stained with DNA fluorochrome PI for 15 min at 37 °C. The samples were immediately analyzed using Facs Calibur flow cytometer (Becton and Dickinson, Heidelberg, Germany).

S4.2.4. Fluorochrome Annexin-V/PI assay for compound 5f

Apoptosis in Huh-7 cells was investigated using fluorescent Annexin V-FITC/ PI detection kit (*BioVision* Annexin V-FITC Apoptosis Detection Kit, Catalog #: K101) by flow cytometry assay. Huh-7 cells at a density of 2×10^5 per well were treated with compound **5f** at the IC_{50} (μM) for 48 h, then the cells were harvested and stained with Annexin V-FITC/ PI dye for 15 min in the dark at 37 °C. The samples were immediately analyzed using *FACS Calibur* flow cytometer (Becton and Dickinson, Heidelberg, Germany).

S4.2.5. Wound healing assay

Huh-7 were grown in 6-well plates for 24 h, and scratches were made using pipette tip and washed with PBS to remove non-adherent cell debris. Subsequently, the cells were treated with naphthalene-enamide analog **5f** for 24 h. The migrations across the wound area were photographed under a phase contrast microscopy.
